# Experimental Study on Flexural Behaviour of Diagonally Notched Wooden Beams Strengthened with Prestressed Superelastic SMA Wires

**DOI:** 10.3390/ma19183918

**Published:** 2026-09-15

**Authors:** Zhongshuai Hu, Ping Lyu, Shaoyuan Zheng, Chunhui Zhang, Liguo Ma

**Affiliations:** 1 School of Energy and Constructional Engineering, Shandong Huayu University of Technology, Dezhou 253034, Chinazch466751195@126.com (C.Z.); 2 Department of Civil, Construction and Environmental Engineering, Iowa State University, Ames, IA 50011, USA; 3 School of Civil Engineering, Yantai University, Yantai 264005, China

**Keywords:** timber structures, SMA wires, flexural performance, Korean pine beams, prestressing

## Abstract

Timber structures constitute the primary form of ancient architecture in China and possess immense historical, artistic and scientific value. Having withstood the ravages of time over hundreds or even thousands of years, timber beams—one of the main load-bearing components in such structures—have largely sustained varying degrees of damage and require repair and reinforcement. Superelastic shape memory alloy (SMA) wires offer numerous advantages, including high strength, corrosion resistance, and stable recovery stress via stress-induced martensitic transformation, and are currently widely used in the field of concrete; however, research into their application for reinforcing timber beams remains limited. This study experimentally investigated the flexural performance of diagonally cracked Korean pine beams reinforced with prestressed Ni–Ti SMA wires. The results demonstrate that SMA reinforcement significantly enhances ultimate load-bearing capacity, with prestressing proving substantially more effective than simply increasing the number of wires. The optimal configuration—two SMA wires at 3% prestress—yielded the greatest improvement of 38.02% in ultimate capacity. Additionally, SMA reinforcement improved flexural stiffness, reduced compressive strain under equivalent loads, and lowered the neutral axis position, thereby enlarging the compression zone. These findings confirm that prestressed superelastic SMA wires offer a highly effective solution for strengthening diagonally cracked timber beams in existing structures.

## 1. Introduction

As a natural, renewable building material, timber plays a significant role in both the conservation of historic buildings and modern timber-frame construction due to its light weight, high strength, good workability and notable environmental benefits [1]. However, as an anisotropic, porous material, the mechanical properties of timber are significantly influenced by factors such as growth defects (knots, wane, etc.), moisture content and the direction of loading [2]. Research has shown that there is an order-of-magnitude difference between the compressive strength of timber along the grain and across the grain, and that most of its mechanical properties increase as the moisture content decreases [3]. During long-term service, the tension zone at the bottom of timber beam members is highly susceptible to cracking damage due to brittle tensile failure of the timber, leading to a significant reduction in the structure’s load-bearing capacity and stiffness [4]. Consequently, the effective reinforcement and repair of damaged timber beams to restore or even enhance their mechanical properties hold significant engineering and academic value.

Researchers have explored a variety of technical approaches for the reinforcement of timber beams. Traditional reinforcement methods include external connections using steel components such as steel plates and bolts, or bonding fibre-reinforced polymer (FRP) composites to the tension zone on the underside of the beam. Relevant experiments have shown that FRP reinforcement can increase the flexural stiffness and ultimate load-bearing capacity of timber beams by 65% and 74%, respectively [5,6,7]. The introduction of prestressing technology has further enhanced the efficiency of reinforcement; by applying prestress to the reinforcement material, the timber beam can undergo reverse deformation in advance under service loads, effectively reducing crack width and increasing the cracking load. Relevant studies indicate that prestressing reinforcement can increase the load-bearing capacity of timber beams by 40.2% and stiffness by 37.9% [8]. However, traditional prestressing systems often involve complex anchorage devices, mechanical tensioning systems, geometric constraints and prestress loss, whilst conventional steel connectors are prone to significant residual deformation under cyclic loading and entail high repair costs; these numerous factors have limited their widespread application in the reinforcement of timber structures [9,10,11].

Superelastic shape memory alloys (SMAs), as a new type of smart material, offer a novel approach to the reinforcement of timber structures due to their unique superelasticity, which enables stress-induced martensitic transformation and full strain recovery upon unloading, providing stable crack-bridging forces under service loads. SMA materials, typified by Ni–Ti alloys, not only possess high strength, high corrosion resistance and excellent fatigue performance, but are also capable of absorbing a large amount of energy under stress through a stress-induced martensitic phase transformation, whilst fully recovering from deformation upon unloading [12]. Superelastic Ni–Ti SMAs can recover up to 8% strain with minimal residual elongation, whilst cyclic loading training can significantly enhance the stability of the SMA wire’s mechanical properties, with a decay rate approaching zero; pre-straining, meanwhile, helps to improve the SMA’s load-bearing capacity and self-resetting ability [13,14,15]. In recent years, research into SMAs in the field of civil engineering has gradually expanded from concrete and steel structures to timber structures. Mirdad and Andrawes conducted a numerical analysis of the application of SMA prestressing technology in the reinforcement of glued-laminated timber beams; the results indicated that this technology can significantly improve the bending stiffness of the beams [10]. With regard to connection joints, research has shown that the energy dissipation capacity of friction joints in glued-laminated timber beams and columns using SMA strips can be increased by approximately 100%; simultaneously, pin-type connectors based on hyperelastic SMA exhibit excellent self-resetting capability and extremely low residual deformation under cyclic loading [16,17]. Furthermore, positive progress has been made in the application of SMA for the reinforcement of traditional timber mortise-and-tenon joints. Although a wealth of research findings has been accumulated regarding the use of SMA in the reinforcement of concrete and steel structures, systematic experimental studies on its application for flexural reinforcement in the tension zone at the bottom of timber beams remain relatively scarce; in particular, quantitative research into the influence of the number of SMA strands and the degree of prestressing on the reinforcement effect is still rare.

Quantitative studies on the influence of the number of SMA strands and the degree of prestressing on the reinforcement effect are still relatively scarce. Against this research background, this paper takes Korean pine beams as the subject of study. Ni–Ti superelastic SMA wires were used to reinforce the bottom tension zone through mechanical prestressing of cracked and damaged beams. Using a four-point bending test system, the study investigated the influence of the number of SMA wires and the degree of prestressing on the ultimate load-bearing capacity, stiffness, deformation behaviour and failure modes of the reinforced beams. Prior to the tests, mechanical property tests—including compressive strength, compressive modulus of elasticity, tensile strength, bending strength, bending modulus of elasticity and shear strength—were conducted on timber from the same batch in accordance with relevant standards. Additionally, cyclic loading and unloading tests, as well as material property tests under different pre-strain conditions, were performed on the SMA wires to obtain the relevant basic mechanical parameters. The aim is to provide experimental evidence and theoretical references for the engineering application of SMA wires in the field of timber structure reinforcement.

## 2. Experimental

### 2.1. Materials

The timber used in this article is Korean pine (*Pinus koraiensis*), supplied by Senxin Timber Co., Ltd. of Liaocheng, Shandong Province China. The material used for reinforcement in this study is Ni–Ti shape memory alloy (SMA) wire.

The nickel–titanium shape memory alloy (SMA) wire used in this study was supplied by Wuhang Metal Materials Co., Ltd., Dongguan, Guangdong, China. Upon receipt, the wire was in a superelastic state. Table 1 summarises the key properties of the shape memory alloy wire, as provided by the manufacturer and verified by tests conducted in this study. It should be noted that the SMA wires were used in their superelastic condition, and no thermal activation was applied during the tests. The reinforcement mechanism is therefore based on superelastic prestressing rather than thermally induced shape memory recovery.

### 2.2. Experimental Plan

In the experimental assessment of Korean pine, test specimens were first prepared in accordance with relevant testing standards. Mechanical property tests were then conducted using a computer-controlled electronic universal testing machine to determine the density, Poisson’s ratio, compressive strength, tensile strength, bending strength, shear strength, as well as the modulus of elasticity in bending and shear, thereby providing the necessary characteristic parameters of the Korean pine for the subsequent bending performance tests on timber beams.

Bending performance tests on reinforced timber beams. In this test, the number of shape memory alloy wires and their pre-strain were varied as independent variables. A total of six reinforced timber beams were designed, and load-bearing performance tests were conducted on these six beams, as well as one intact timber beam and one cracked timber beam. The study investigated reinforcement methods using shape memory alloy wires at the bottom of timber beams and proposed a novel reinforcement approach. Through testing of the intact timber beam, the cracked timber beam and the reinforced timber beams, relevant data such as the ultimate load-bearing capacity, stress and strain of each timber beam were obtained. Origin software (https://www.originlab.com/) was used to plot load–deflection and load–strain curves for the different timber beams. A comparative analysis was conducted to assess the improvement in mechanical performance indicators—including ultimate load-bearing capacity, stress and strain—of the timber beams under different reinforcement methods.

### 2.3. Test Methods for the Mechanical Properties of Timber and the Parameters Obtained

#### 2.3.1. Moisture Content and Density

**Figure 1:**
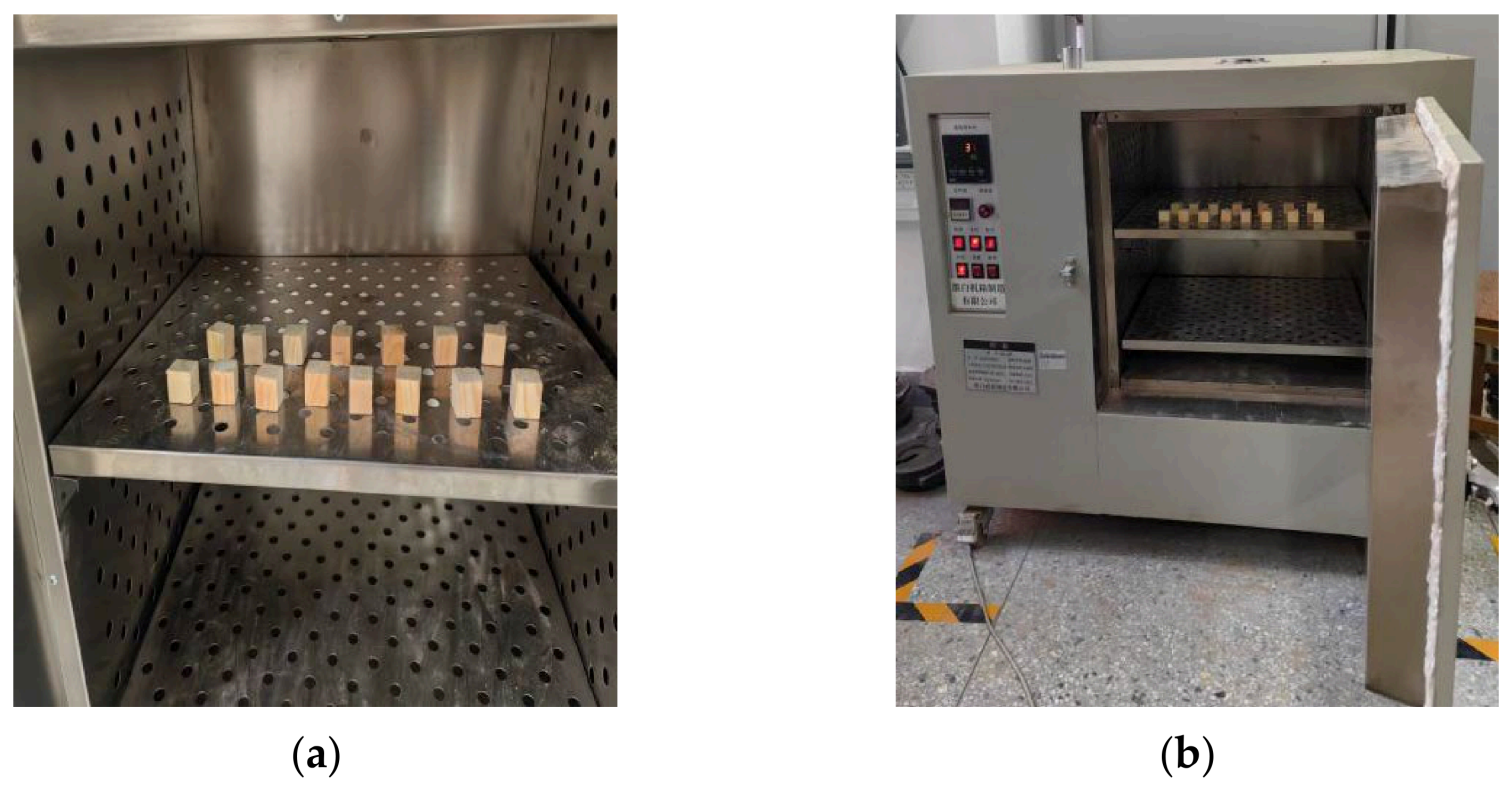
Moisture content test. (**a**) Drying small samples. (**b**) Electric forced-air drying oven.

To minimise the influence of moisture content on the timber, the moisture content of the small test specimens used in each mechanical property test was determined immediately after the completion of that test, in order to obtain the timber’s moisture content and allow for the correction of the test results. Specimens were cut in accordance with standards [18,19]. First, the numbered specimens were weighed on an electronic balance and the readings recorded. The specimens were then placed in an electric forced-air oven for drying and weighed at specified intervals until they reached a fully dry state. All specimens were removed, cooled to room temperature, weighed again, and the results recorded. The test procedure is illustrated in Figure 1. Calculations were performed using Equations (1)–(3) to determine the moisture content and density of the specimens, where *W* is the moisture content of the specimen (%); *m*_1_ is the mass of the specimen before drying (g); *m*_0_ is the absolutely dry mass of the specimen (g); *ρ_w_* is the density of the specimen at the time of testing (g/cm^3^); *m_w_* is the mass of the specimen at the time of testing (g); *V_w_* is the volume of the specimen at the time of testing (cm^3^); *ρ*_0_ is the absolute dry density of the specimen (g/cm^3^); m_0_ is the absolute dry mass (g); and *V*_0_ is the absolute dry volume (cm^3^).

(1) $$W=\frac{m_{1}-m_{0}}{m_{0}}\times 100\%$$

(2) $$\rho_{w}=\frac{m_{w}}{v_{w}}$$

(3) $$\rho_{1}=\frac{m_{1}}{v_{1}}$$

To ensure the reliability of the mechanical property data, the moisture content of each defect-free small specimen was determined immediately after each test, using the oven-drying method specified in GB/T 1927.4-2021 [19]. The results of the moisture content measurements for each type of mechanical property test are summarised in Table 2. As shown in the table, the measured moisture content of all specimens ranged from 10.3% to 12.6%, with an overall average of 11.4%. All individual measurements fell within the applicable range of 9% to 15% specified in the relevant GB/T standards (GB/T 1927.11–1927.16).

#### 2.3.2. Compressive Strength

**Figure 2:**
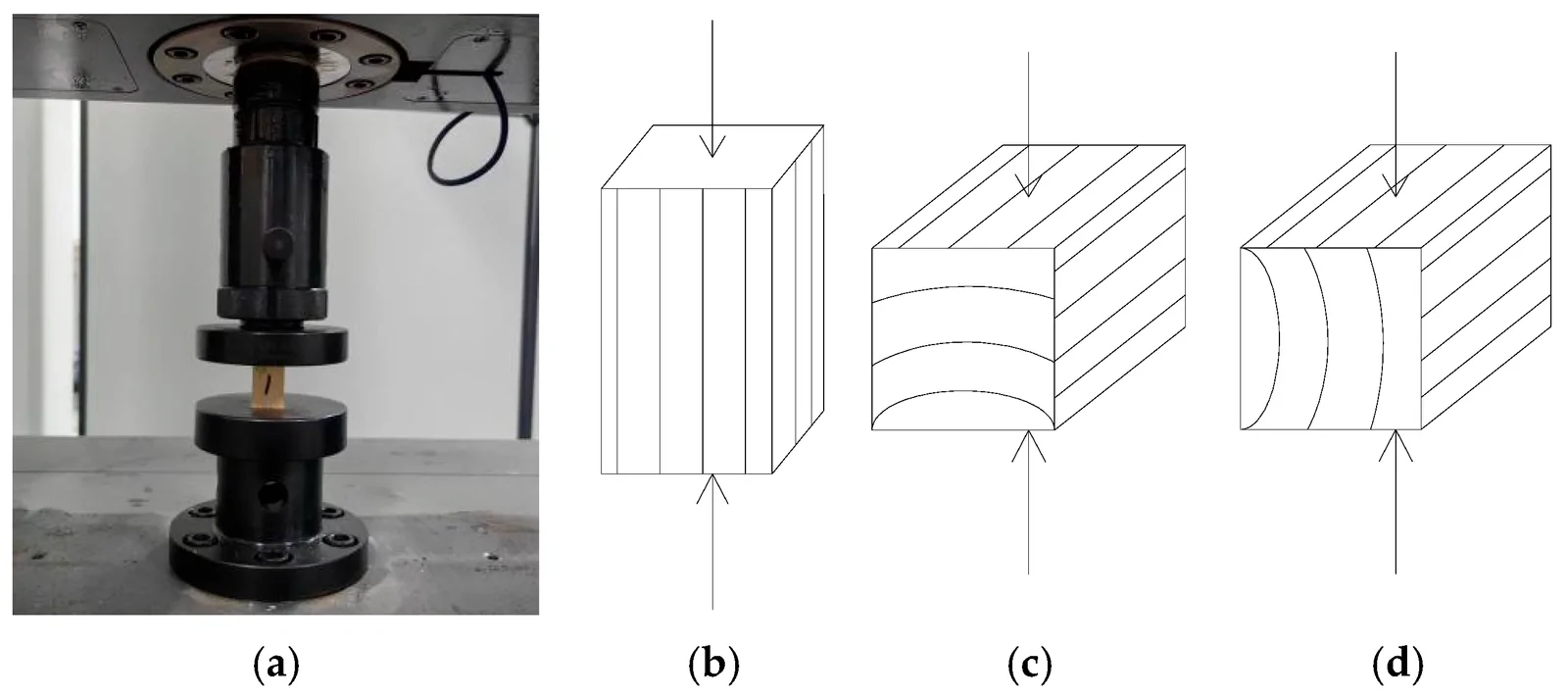
Compressive strength test. (**a**) Compression test along the grain. (**b**) Compressive strength along the grain. (**c**) Transverse radial compression. (**d**) Transverse compressive strength.

In order to determine the compressive strength of the timber selected for this study, and in accordance with Parts 11 [20] and 12 [21] of the Test Methods for Physical and Mechanical Properties of Flawless Small Timber Specimens, six test specimens each were selected from the same batch of test beams for the long-grain compressive strength, cross-grain radial compressive strength and cross-grain tangential compressive strength tests. A computer-controlled electronic universal testing machine was used to conduct the loading tests. In accordance with the specifications, the load was increased at a uniform rate to cause failure of the specimens within 1.5 to 2 min. The test process is illustrated in Figure 2. The test data were averaged, organised and summarised, as shown in Table 3, Table 4 and Table 5. The corrected compressive strength of the timber in the grain direction is greater than that in the cross-grain direction, being approximately nine times the latter, after moisture content adjustment to 12% MC.

After the specimen has failed, its moisture content is determined in accordance with the standard. When the moisture content of the timber is between 9% and 15%, the compressive strength along the grain, the compressive strength across the grain in the radial direction, and the compressive strength across the grain in the tangential direction are corrected using Equations (4) and (5) respectively, and converted to strength values corresponding to a moisture content of 12%.

(4) $$\sigma_{c,12}=\sigma_{w}\left[1+0.05\left(W-12\right)\right]$$

(5) $$\sigma_{y,12}=\sigma_{y}\left[1+0.045\left(W-12\right)\right]$$

#### 2.3.3. Compressive Elastic Modulus and Poisson’s Ratio

**Figure 3:**
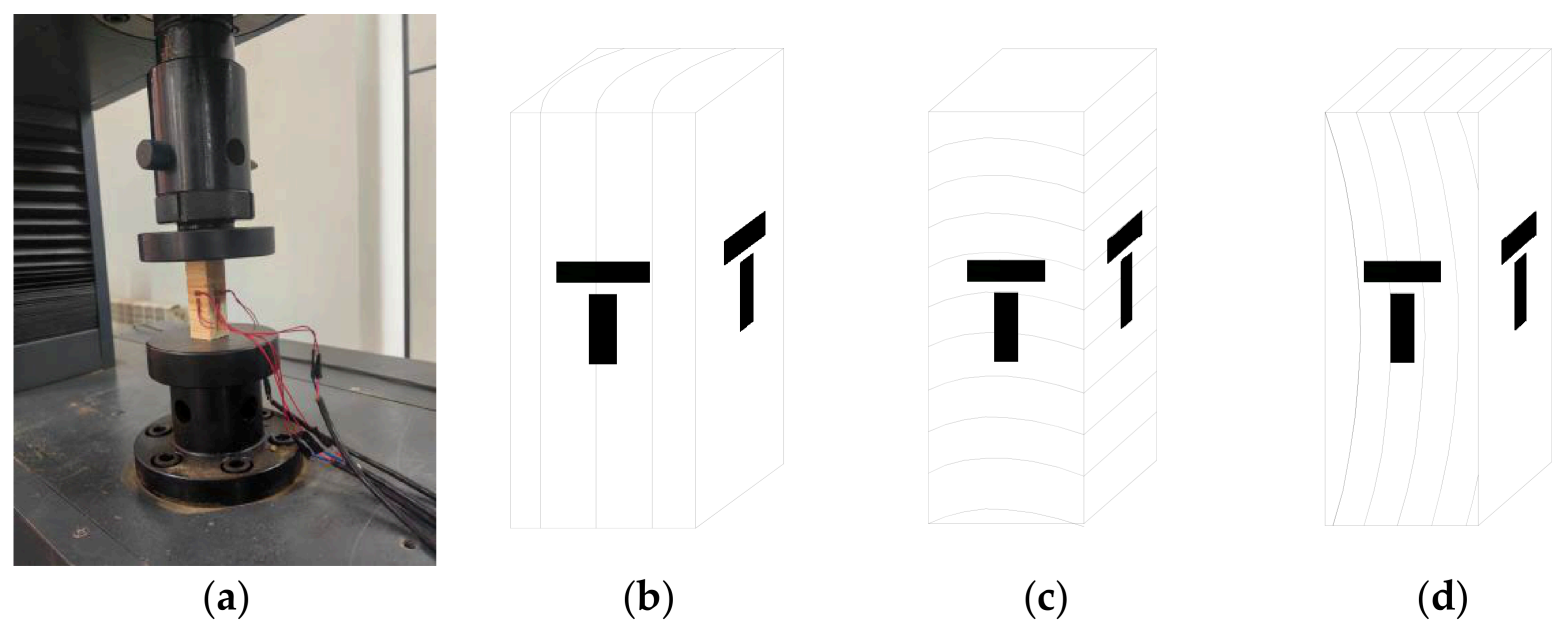
Compressive elastic modulus test. (**a**) Compressive modulus of elasticity in the grain direction. (**b**) Grain direction. (**c**) Transverse radial direction. (**d**) Transverse tangential direction.

The compressive modulus of elasticity of the Korean pine selected for this study was determined in accordance with standard [22]. From the same batch of test timber beams, six test specimens were selected for each of the following: compressive elastic modulus in the grain direction, compressive elastic modulus in the radial direction, and compressive elastic modulus in the tangential direction. All specimens measured 60 mm × 20 mm × 20 mm. To determine the elastic modulus and Poisson’s ratio of the specimens, strain gauges were affixed to the sides of the specimens, and testing was carried out using a universal testing machine. In accordance with the specifications, the load range was set between 1000 N and 4000 N, with the loading and unloading cycle repeated six times. The test procedure is illustrated in Figure 3. The experimental data were averaged, organised and summarised, as shown in Table 6, Table 7 and Table 8.

To minimise the effect of moisture content on the mechanical properties of timber, when the moisture content of the test specimens is between 9% and 15%, the compressive elastic modulus in the longitudinal direction, the compressive elastic modulus in the transverse radial direction and the compressive elastic modulus in the transverse tangential direction may be corrected using Equations (6) and (7) respectively.

(6) $$E_{12}=E_{w}\left[1+0.012\left(W-12\right)\right]$$

(7) $$E_{12}=E_{w}\left[1+0.055\left(W-12\right)\right]$$

The transverse and longitudinal strains of the specimen were also recorded during the compressive elastic modulus test. However, during data analysis, the calculated Poisson’s ratios exhibited considerable scatter and included values that were not physically reasonable for softwood (e.g., values approaching 0.9). These anomalies are likely attributable to measurement errors in the strain gauge bonding or data acquisition. Since these Poisson’s ratio data were not used in any subsequent analysis or calculation, they are omitted from this paper. For the calculation of shear moduli presented in Section 2.3.8, the standard relationship G ≈ E/[2(1 + ν)] was not adopted; instead, the shear moduli were estimated using typical relationships for orthotropic softwood reported in the literature.

#### 2.3.4. Tensile Strength

**Figure 4:**
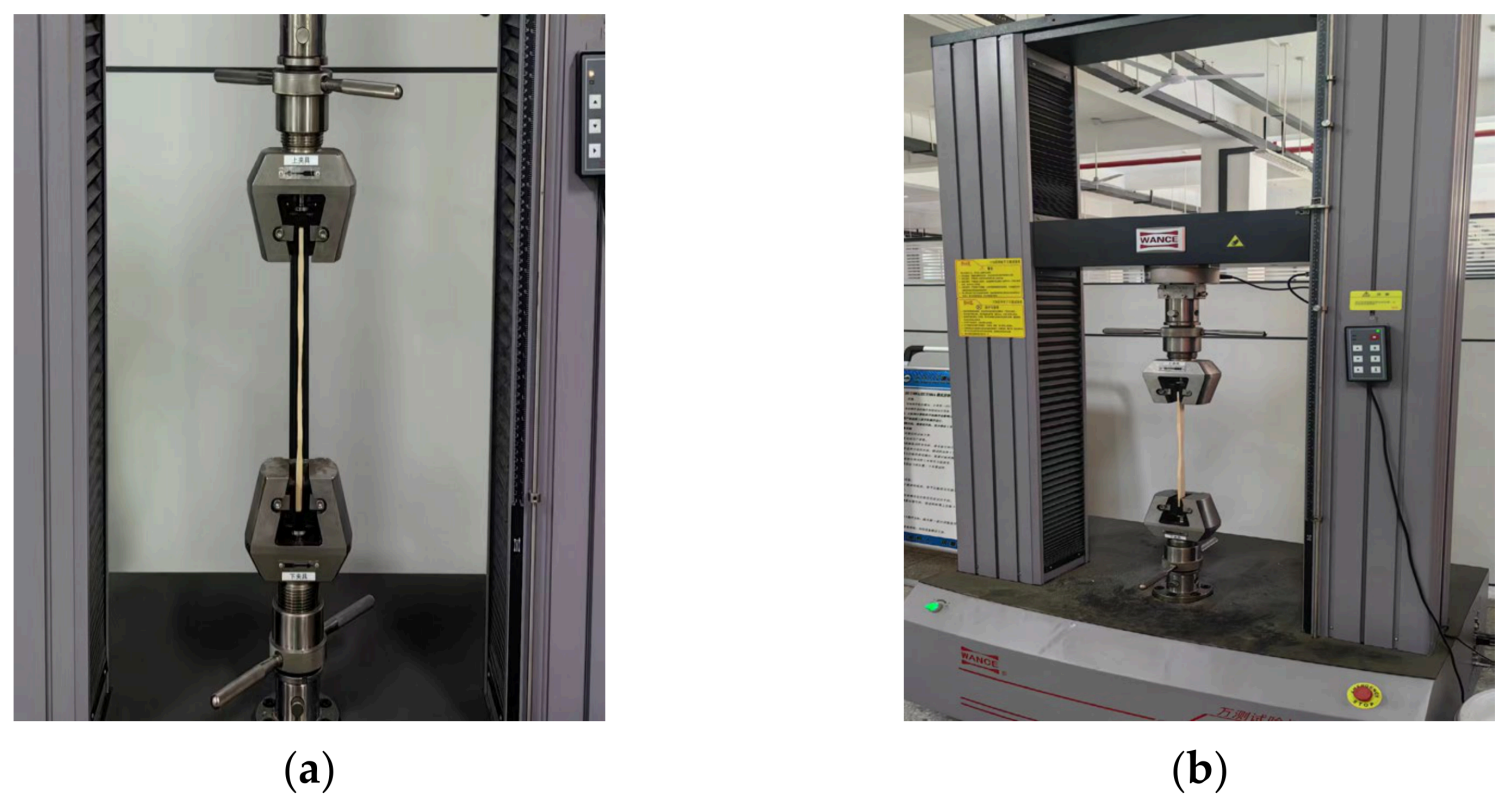
Tensile strength test. (**a**) Tensile strength test along the grain. (**b**) Tensile strength testing equipment.

In accordance with standard [23], six specimens each for longitudinal tensile strength and six for transverse tensile strength were prepared. The specimens were mounted in the appropriate positions on the universal testing machine in accordance with the specifications, and a load was applied at a uniform rate to cause failure of the specimens within 1.5 to 2 min. The test procedure is shown in Figure 4. Specimens that had fractured within the effective section were selected, and the width and thickness at the point of failure were measured using a vernier calliper. The test results and measurement data are recorded in Table 9, Table 10 and Table 11. The test results show that the tensile strength of the wood along the grain is greater than its compressive strength along the grain, whilst the tensile strength across the grain is significantly lower than that along the grain.

To measure tensile strain, two electrical resistance strain gauges (model BX120-3AA, gauge factor 2.08 ± 1%) were symmetrically bonded to the mid-length of each tensile specimen on opposite faces. The gauge length adopted was 50 mm, in accordance with GB/T 1927.14-2022. The strain gauges were connected to a DH3816N static strain data acquisition system with a sampling frequency of 1 Hz. Prior to each test, all strain readings were zeroed. Strain values were recorded continuously throughout loading, providing direct measurements of specimen deformation without interference from machine compliance or grip slip, since the gauges were bonded directly to the timber surface. The reported tensile strain values are the average of the readings from the two strain gauges at the maximum load, calculated as engineering strain ε = ΔL/L_0_. Crosshead displacement was monitored only for reference and was not used in strain calculations.

After the test, samples shall be taken from the valid section for moisture content testing. Where the moisture content is between 9% and 15%, the tensile strength in the grain direction shall be corrected using Equation (8), and the tensile strength across the grain shall be corrected using Equation (9). The results of the corrections are shown in Table 8, Table 9 and Table 10.

(8) $$\sigma_{t,12}=\sigma_{w}\left[1+0.015\left(W-12\right)\right]$$

(9) $$\sigma_{t,12}=\sigma_{w}\left[1+0.025\left(W-12\right)\right]$$

#### 2.3.5. Modulus of Elasticity in Bending

**Figure 5:**
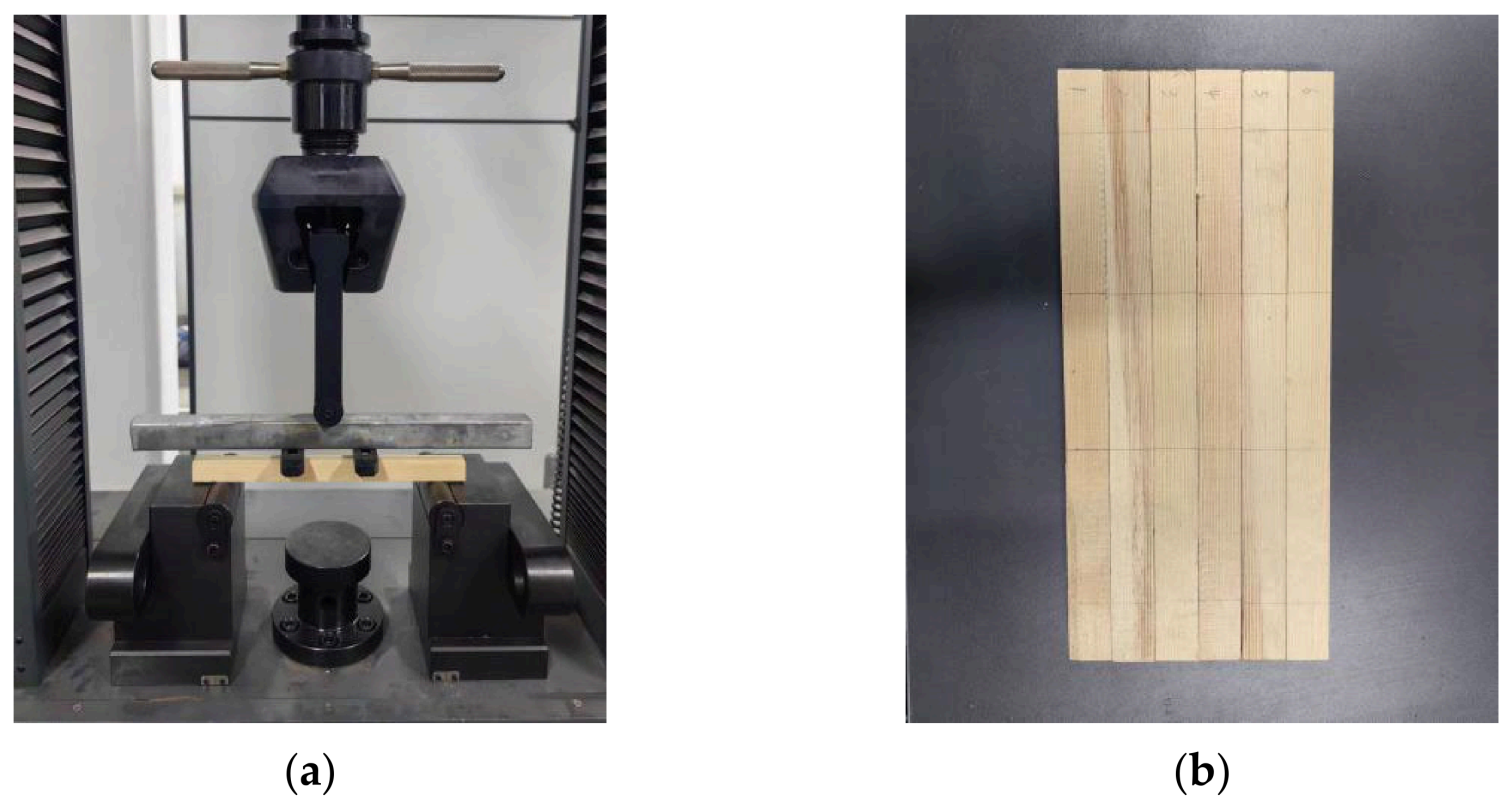
Test for the modulus of elasticity in bending. (**a**) Modulus of elasticity in bending. (**b**) Test specimen for the modulus of elasticity in bending.

Specimens for determining the flexural modulus of elasticity were designed in accordance with standard [24], with dimensions of 300 mm × 20 mm × 20 mm, the length being in the grain direction. The specimens were subjected to loading tests at a uniform rate; the strain values were recorded when the load reached the lower limit, and again when it reached the upper limit, after which the load was unloaded. This cycle was repeated four times; the test procedure is illustrated in Figure 5. The difference between the average deformation values at the lower and upper load limits from the last three test cycles was taken as the specimen deformation values at the lower and upper load limits for the calculation of the flexural modulus of elasticity. The results of the correction are shown in Table 12.

Once the measurement of the modulus of elasticity in bending has been completed, the same specimen is used to conduct a bending strength test. After both tests have been completed, a 20 mm long section is cut from the centre of the specimen for moisture content testing. To minimise the effect of moisture content on the modulus of elasticity in bending, when the specimen’s moisture content is between 9% and 15%, the modulus of elasticity in bending is corrected using Equation (10).

(10) $$E_{12}=E_{w}\left[1+0.015\left(W-12\right)\right]$$

#### 2.3.6. Flexural Strength

**Figure 6:**
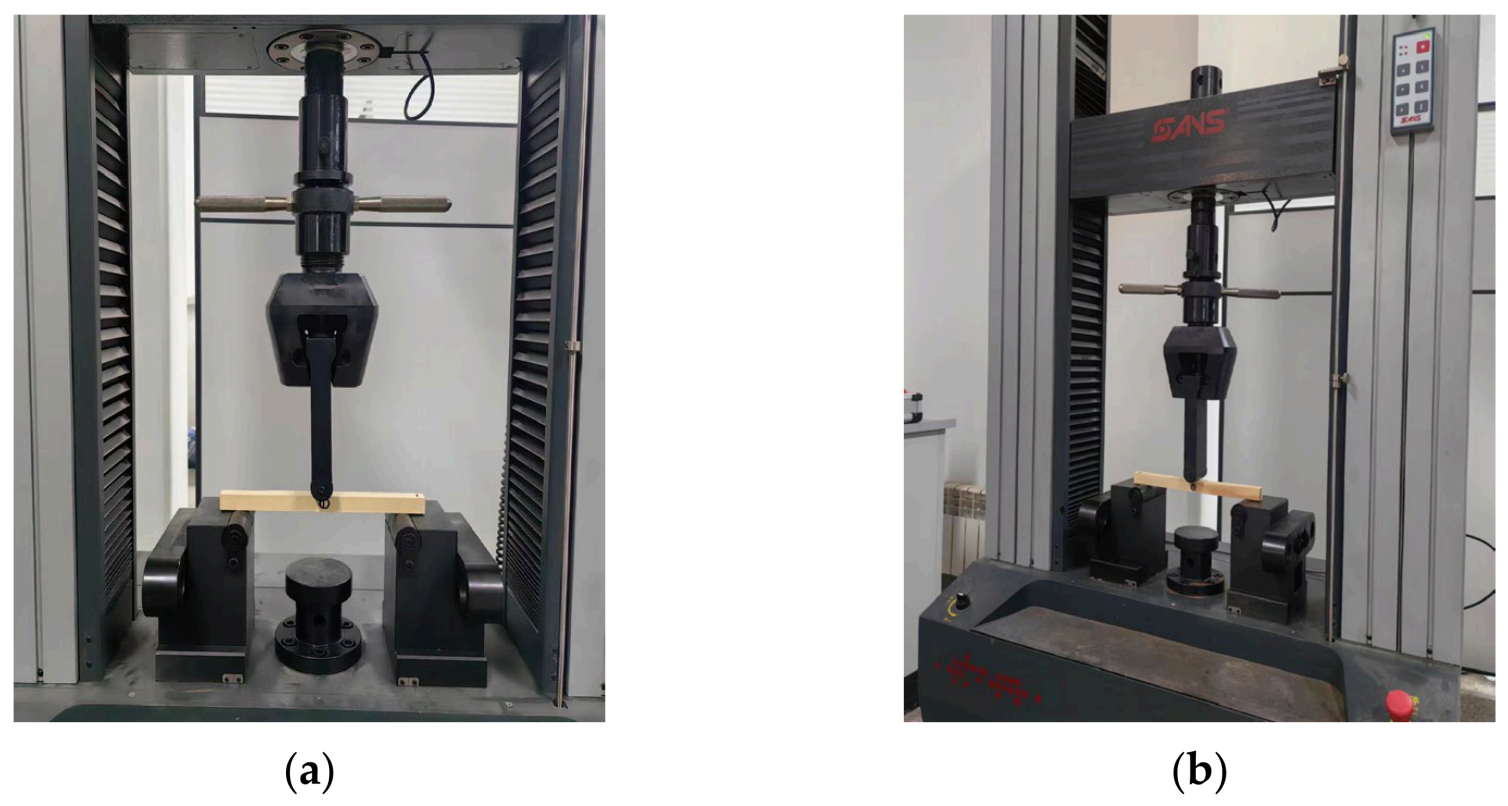
Flexural strength test. (**a**) Flexural strength. (**b**) Flexural strength testing equipment.

In accordance with standard [25], a flexural strength test was conducted on the same specimen used for the flexural modulus of elasticity test. First, the dimensions of the specimen were measured using a vernier calliper. The specimen was then placed on the two supports of the universal testing machine, and a central load was applied along the chord at a uniform rate, causing the specimen to fail within 1 to 2 min. The test procedure is shown in Figure 6, and the failure load was recorded. The test results are shown in Table 12.

Immediately after the test, cut the specimens required for determining the moisture content of the test sample and carry out the moisture content measurement in accordance with the specified procedure, in order to correct for the effect of moisture content on the bending strength of the timber. When the moisture content of the specimen is within the range of 9% to 15%, the bending strength shall be corrected in accordance with Equation (11). The test results and corrections are shown in Table 13.

(11) $$\sigma_{b,12}=\sigma_{b,w}\left[1+0.04\left(W-12\right)\right]$$

#### 2.3.7. Shear Strength

**Figure 7:**
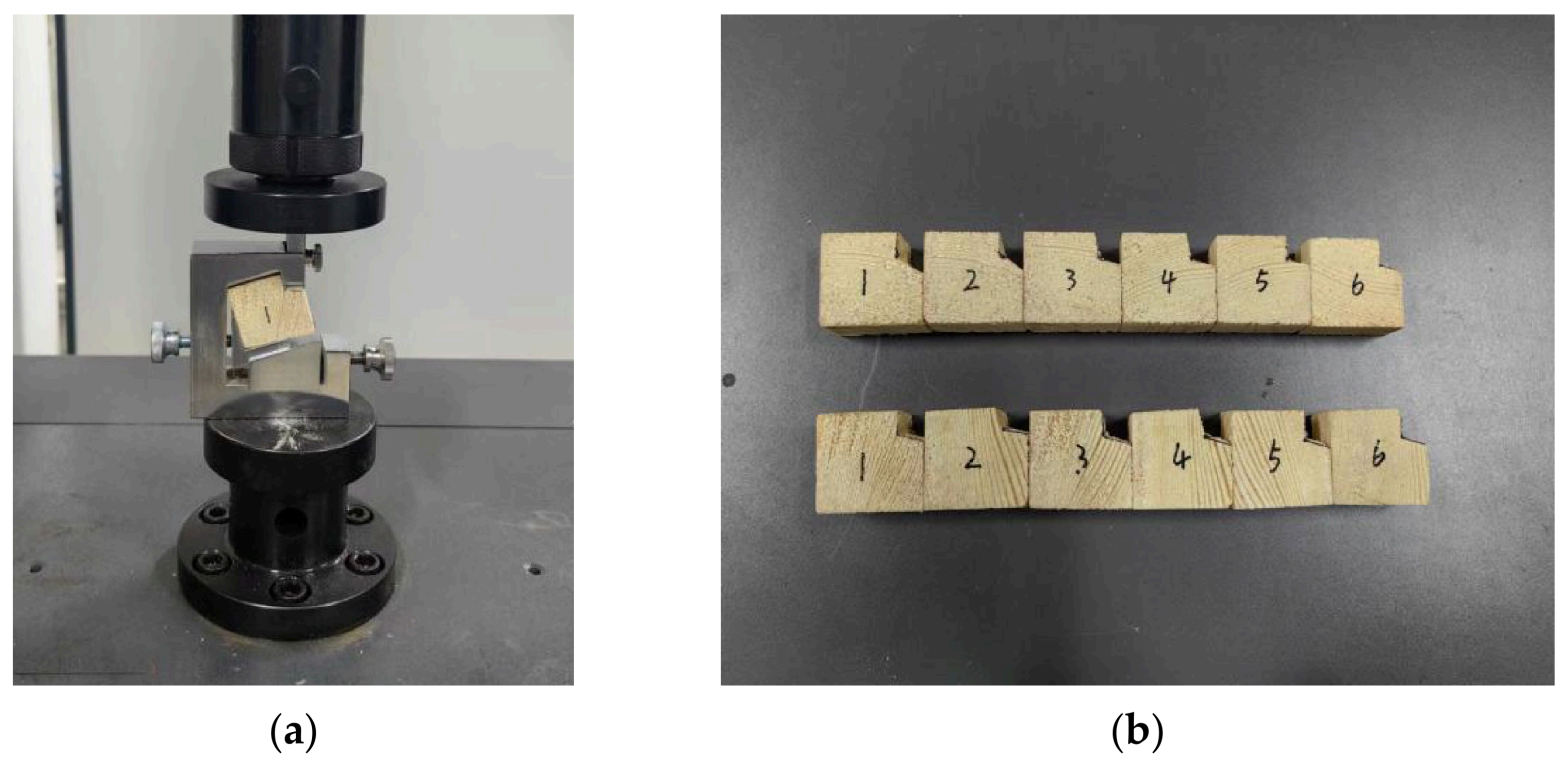
Shear strength test. (**a**) Shear strength. (**b**) Shear strength test specimen.

Shear strength test specimens were prepared in accordance with standard [26], comprising six specimens each for the tangential and radial directions, all with lengths aligned with the grain direction; the shear surfaces of the specimens were either radial or tangential. Prior to testing, measure the dimensions of the shear surfaces of the specimens using a vernier calliper and check the angles of the notched sections using a protractor. Then, place the specimens in the shear testing apparatus, ensuring they fit snugly against the apparatus without any wobbling. Mount the apparatus with the specimens in place onto the universal testing machine and apply the load at a uniform rate; the testing procedure is illustrated in Figure 7. Upon completion of the test, record the dimensions of the shear surface and the failure load, and calculate the shear strength in the grain direction for the chord or radial surface using the formula. The test results are shown in Table 14 and Table 15.

The moisture content of the small fragments remaining after the specimen has been broken is determined, and corrections are applied in accordance with Equations (12) and (13) to minimise the influence of moisture content on the shear strength. The results of the corrections are shown in Table 14 and Table 15.

(12) $$\tau_{w}=\frac{0.96P_{\max}}{bl}$$

(13) $$\tau_{12}=\tau_{w}\left[1+0.03\left(W-12\right)\right]$$

It should be noted that the shear area A in Equations (13) and (14) refers to the actual shear area, i.e., the product of the specimen width b and the length l of the shear plane measured along the grain direction (A = b × l), as specified in GB/T 1927.16-2022 [26]. The cross-sectional area of the specimen (20 mm × 20 mm) shall not be used in the calculation. The length l of the shear plane shall be measured prior to the test using a vernier calliper, accurate to 0.1 mm. The maximum failure load Pmax shall be recorded during the test, and the shear strength at the test moisture content shall be calculated as τ = P_max_/(b × l). The moisture content of each specimen was determined immediately after the test in accordance with GB/T 1927.4-2021, and the strength was then corrected to a moisture content of 12% using Equations (13) and (14). The corrected results are shown in Table 14 and Table 15.

#### 2.3.8. Shear Modulus

The shear modulus of timber is related to its compressive moduli of elasticity in the longitudinal (E), radial (E_r_), and tangential (E_t_) directions. For an orthotropic material, the shear moduli can be derived from the engineering constants. However, given that the measured Poisson’s ratios in this study were unreliable (as discussed in Section 2.3.3), the standard relationship G ≈ E/[2(1 + ν)] was not used. Instead, the shear moduli were estimated using the following typical relationships for softwood reported in the literature [27]: G_rl_ ≈ E_l_/16, G_tl_ ≈ E_l_/18, and G_rt_ ≈ E_r_/8. Based on the measured elastic moduli (E_l_ = 9898.3 MPa, E_r_ = 1521.5 MPa, E_t_ = 762.6 MPa), the calculated shear moduli are presented in Table 16.

#### 2.3.9. Mechanical Properties of Ni–Ti SMA Wire

**Figure 8:**
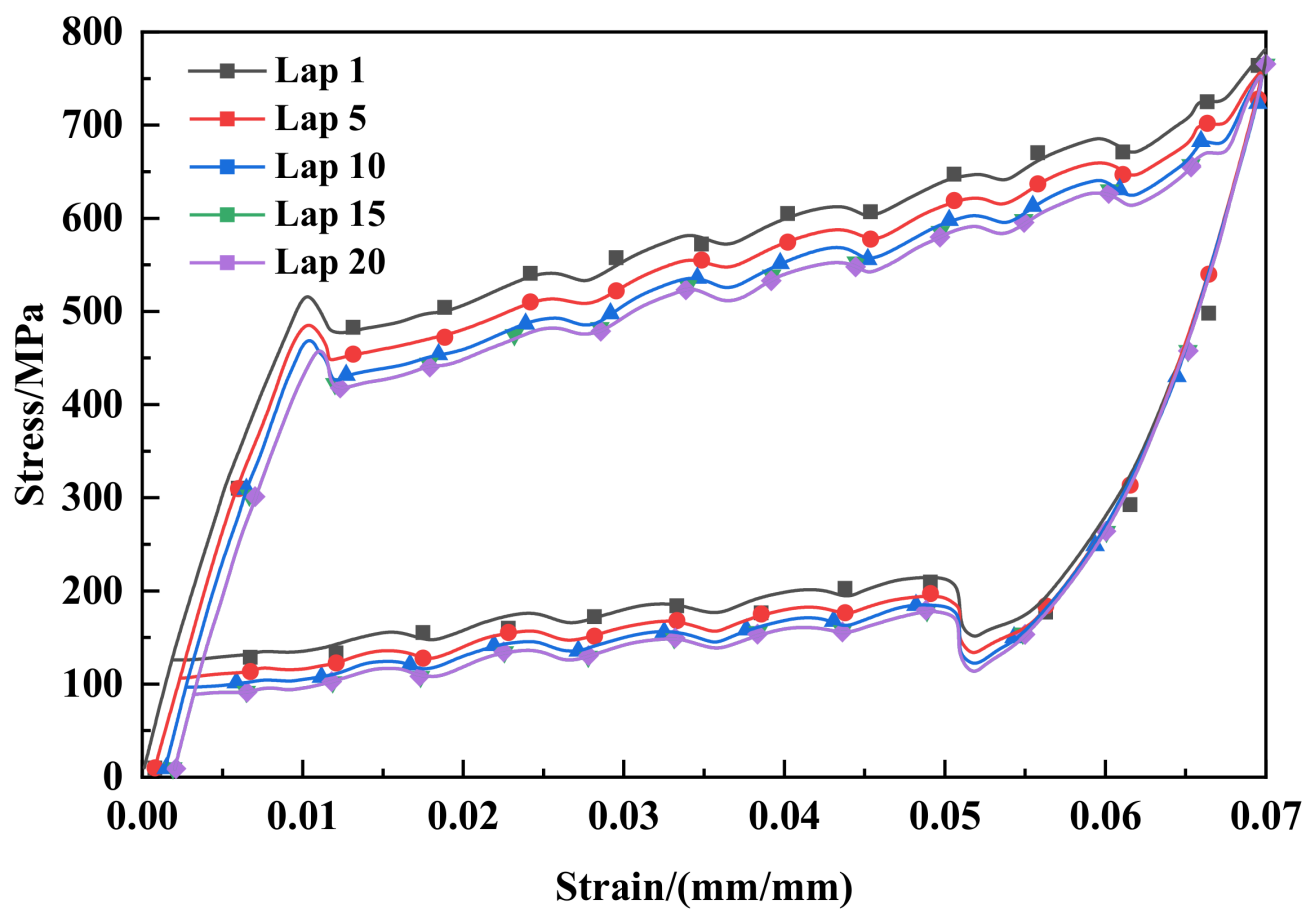
Stress–strain curves of SMA wire under different numbers of cyclic loads.

To investigate the effects of cycle count and pre-strain on the mechanical properties of Ni–Ti SMA wires, superelasticity tests were conducted on the SMA wires. Specifically, two 150 mm SMA wire specimens were selected and subjected to 20 cycles of loading and unloading at room temperature, with a loading rate of 1% per minute and a strain amplitude of 7%. Figure 8 was plotted based on the test results, showing the engineering stress and engineering strain curves for the SMA wire at the 1st, 5th, 10th, 15th and 20th cycles of loading and unloading. This test served to stabilise the material properties; all subsequent tests utilised the wires following this conditioning process. These tests confirm the superelastic behaviour of the wires at room temperature. The stable stress–strain response after cyclic conditioning (Figure 8) demonstrates the wire’s ability to provide a sustained recovery stress after mechanical prestressing, without the need for thermal activation.

**Figure 9:**
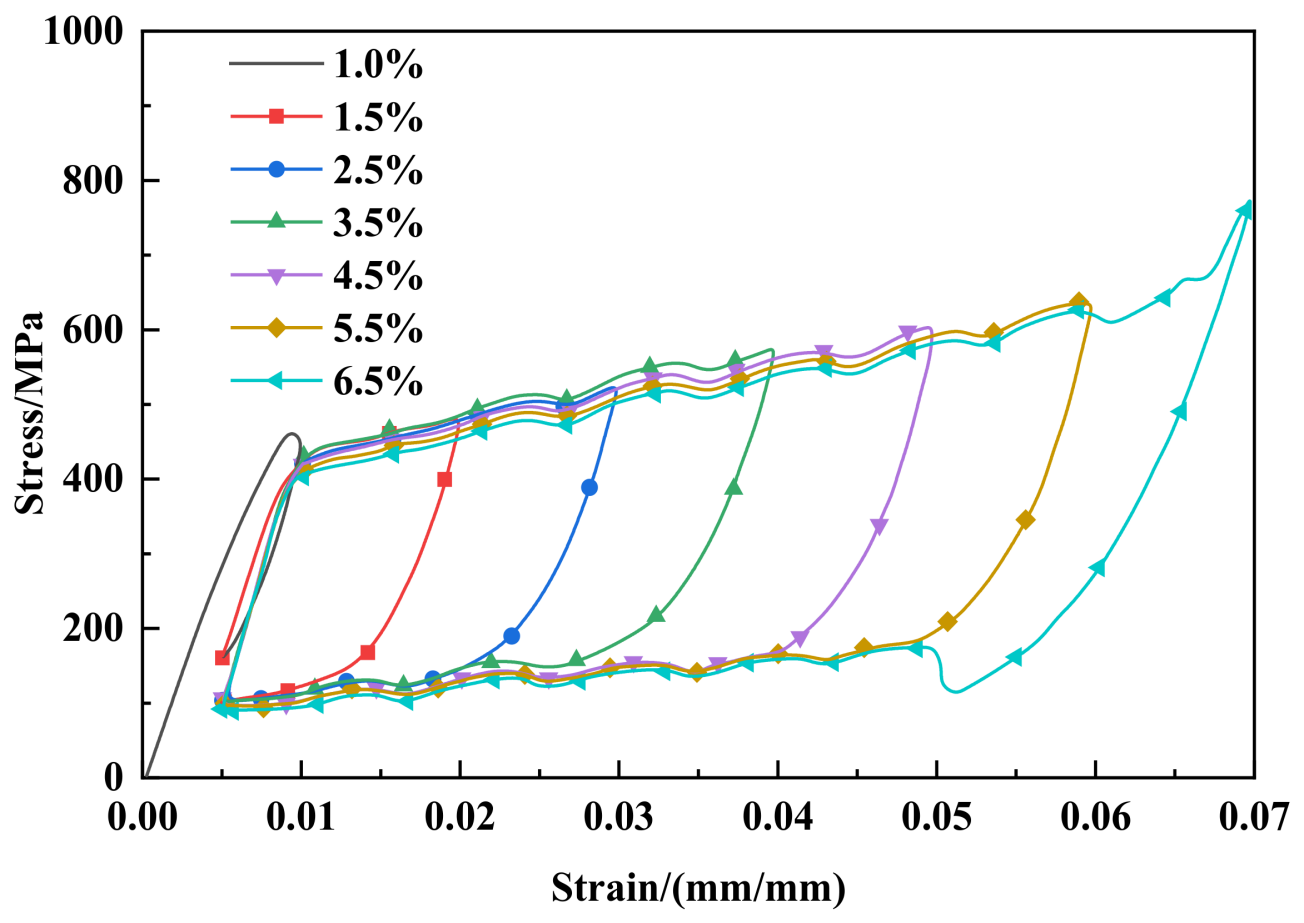
Stress–strain curve of SMA wire at 0.5% initial strain.

To investigate the effect of prestressing on the mechanical properties of Ni–Ti SMA wires, loading and unloading tests were conducted on prestressed wires at room temperature. The initial strain of the SMA wire used in the tests was 0.5%, and the strain loading profile was 0 → 1% → 0.5% → 2% → 0.5% → 3% → 0.5% → 4% → 0.5% → 5% → 0.5% → 6% → 0.5% → 7% → 0.5%. The test results are shown in Figure 9.

### 2.4. Test Method for the Load-Bearing Performance of SMA-Reinforced Timber Beams

#### 2.4.1. Test Conditions

A total of eight timber beams of identical dimensions were designed for this experiment; all were made of Korean pine (*Pinus koraiensis*) and had rectangular cross-sections. Research conducted by the project team revealed that cracks in timber beams within timber structures typically extend upwards from the bottom of the beam. The team members investigated the flexural performance of cracks at different locations and angles; based on these findings, this paper selected the most unfavourable crack location and angle for reinforcement studies. Consequently, artificial notches (machined slots) were introduced into both the cracked timber beams and the reinforced timber beams to simulate a cracked condition. All cracks originated from the centre point at the bottom of the right-hand shear-bending section and extended towards the mid-span at a 6° angle. Specimen L1-1 was an intact timber beam; specimen L2-1 was a cracked timber beam without SMA wire reinforcement; specimens L3-1, L4-1 and L5-1 constituted the SMA wire quantity comparison group, reinforced with 1, 2 and 3 SMA wires respectively; and specimens L6-1, L7-1 and L8-1 constituted the SMA wire prestressing comparison group, with SMA wire prestressing levels of 1%, 2% and 3% respectively. The specific specimen dimensions and reinforcement methods are shown in Table 17.

It is important to note that in aged timber structures, diagonal cracks typically exhibit irregular fracture surfaces, interlocking fibres and varying degrees of micro-damage along the crack path; these factors may all significantly influence local stress redistribution and crack propagation behaviour, whilst artificially induced cracks may not perfectly replicate all the complex characteristics of naturally aged cracks. The decision to use artificially induced cracks in this study was based on the following considerations. Firstly, to ensure reproducibility and comparability between different reinforcement schemes, it is essential to standardise the initial cracking conditions; however, precise control of the geometric parameters of cracks is extremely difficult to achieve in naturally formed or aged cracks. Secondly, the specific crack dimensions (190 mm long, 10 mm wide, 20 mm deep) and the 6° inclination angle were determined based on the research team’s previous field surveys of typical damage patterns in historic timber beams, ensuring that the artificially created cracks represent the most unfavourable cracking conditions commonly encountered in engineering practice. Thirdly, the primary objective of this study is to evaluate the relative improvement in flexural performance (ultimate load-bearing capacity, stiffness and strain distribution) achieved through SMA wire reinforcement, rather than to replicate every detail of a real crack; the artificial crack therefore provides a clear and consistent benchmark for this comparative evaluation. However, in actual engineering applications, the effectiveness of the reinforcement may be influenced by the specific morphology of real cracks. Nevertheless, the general conclusions regarding the number of SMA wires and the level of prestressing are expected to remain valid in qualitative terms, as these factors primarily influence the overall flexural behaviour of the SMA wires and their crack-bridging effect, rather than local details on the crack surface.

Furthermore, in this study, all ‘defect’ specimens were prefabricated with rectangular slots (slots/notches) cut using a power saw, each 10 mm wide. The horizontal projection length of the slot on the underside of the beam was 190 mm, with a depth of 20 mm and a width of 10 mm; it was inclined at an angle of 6° (relative to the underside of the beam) and extended obliquely from the centre point of the underside of the right-hand bent section towards the mid-span. The starting point of the slot was situated between the right-hand loading point and the right-hand support, approximately 300 mm from the centreline of the right-hand support, whilst the end point was located near the centre of the span. These geometric parameters were derived from the research group’s previous field surveys of typical damage in historic timber structures and represent the most unfavourable form of oblique cracking encountered in actual engineering practice. Compared with real aged oblique cracks, the saw-cut notch completely severs the wood fibres, eliminating fibre bridging and interlocking effects, and results in a higher degree of stress concentration; it therefore represents a conservative simulation condition.

#### 2.4.2. Reinforcement Plan for Test Specimens

**Figure 10:**
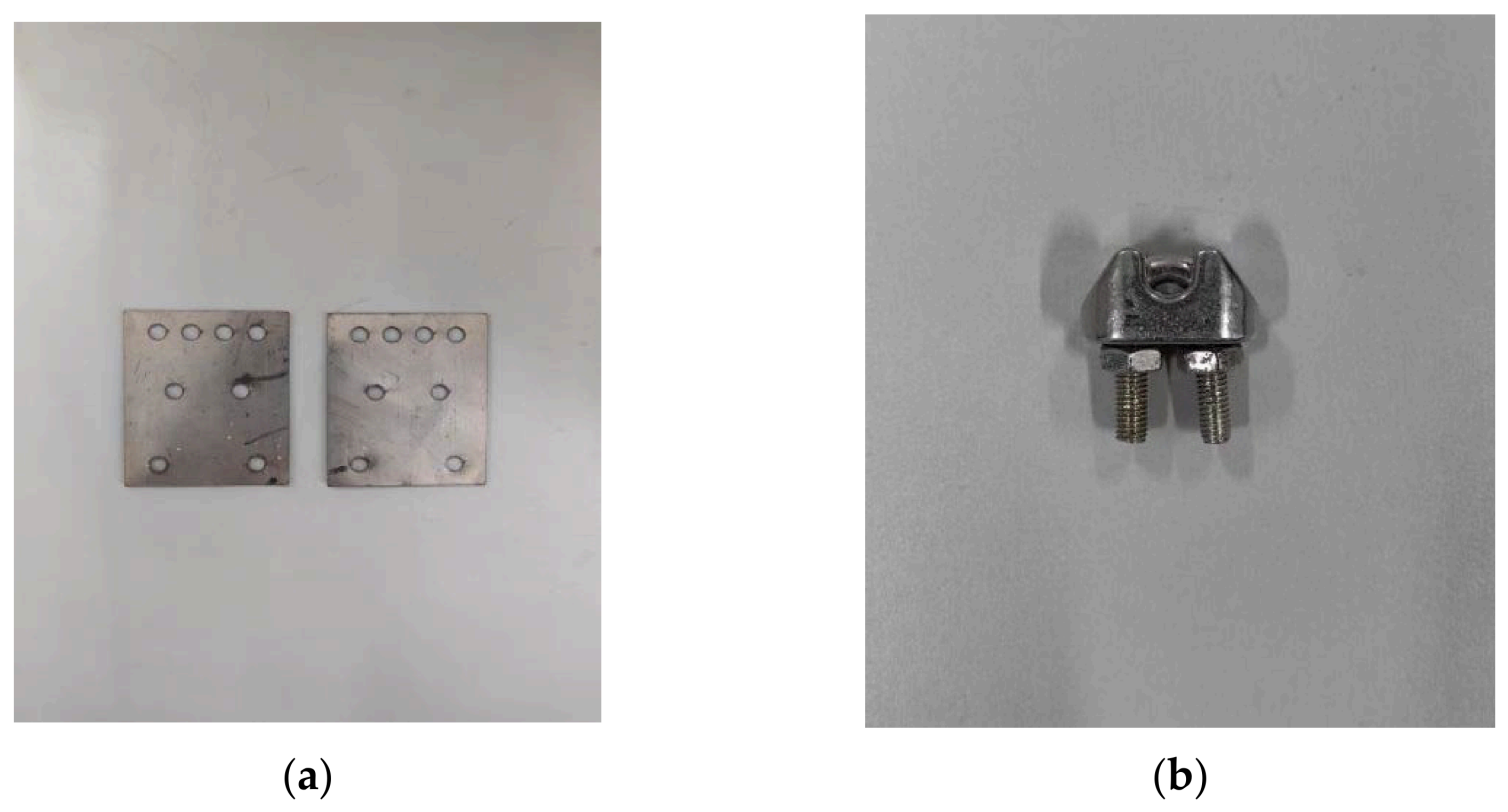
SMA wire fixing assembly. (**a**) Stainless steel plate securing the end of the SMA wire. (**b**) SMA wire retaining clip.

**Figure 11:**
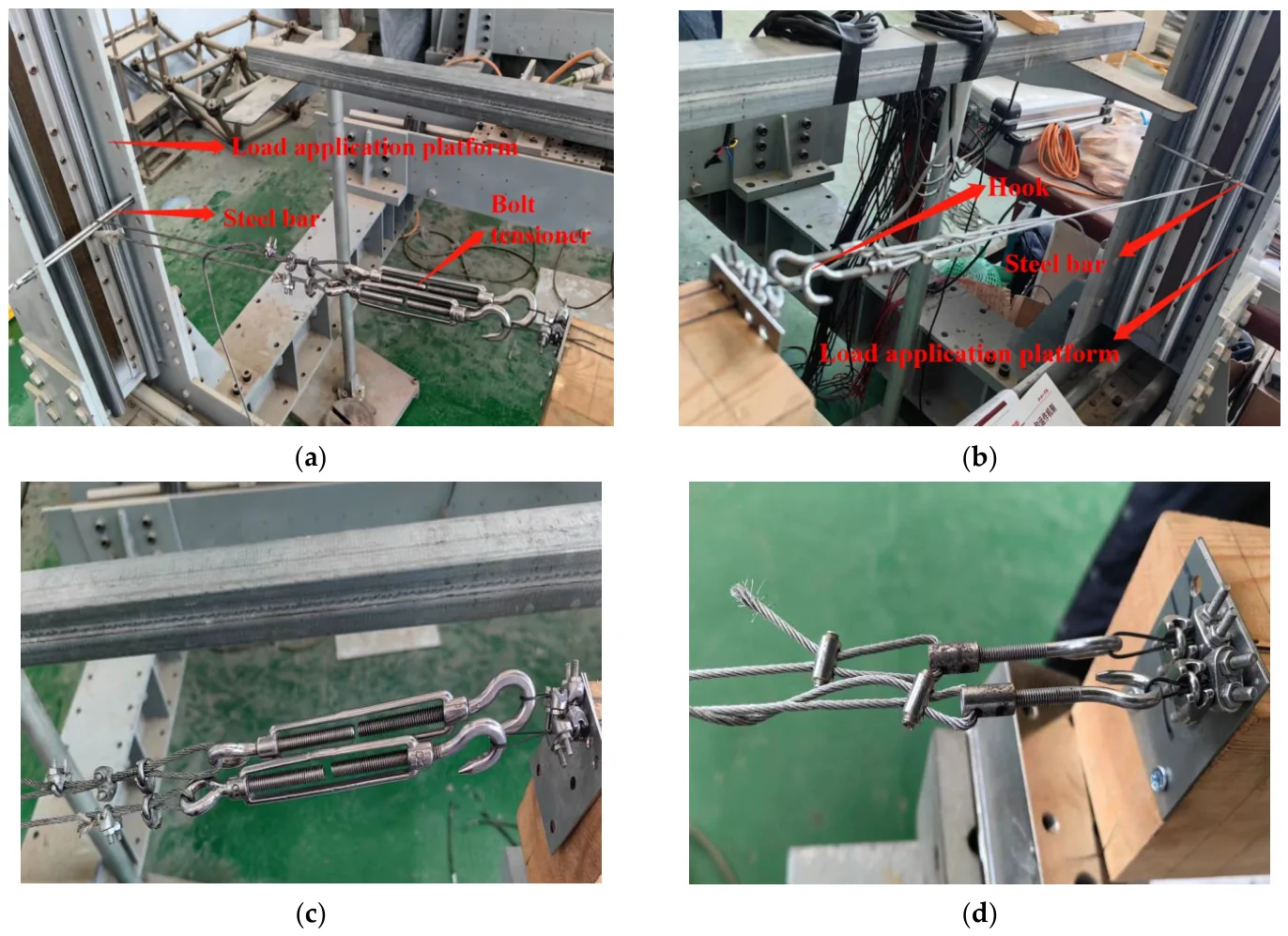
SMA wire tensioning device. (**a**) Left-hand tensioning device. (**b**) Right-hand tensioning device. (**c**) SMA threaded bolt tensioner. (**d**) SMA cable connection hook.

This test employs a novel SMA wire reinforcement method to reinforce the timber beam. The SMA wire fixing assembly, shown in Figure 10, comprises custom-made stainless steel plates and clips at both ends of the timber beam. The SMA wire tensioning assembly, shown in Figure 11, comprises steel rods fixed at both ends to the loading platform, two hooks on the right-hand side, and two bolt tensioners on the left-hand side. It should be noted that the prestressing procedure described herein is purely mechanical, achieved by bolt tensioners; no electrical or thermal activation was applied to the SMA wires.

**Figure 12:**
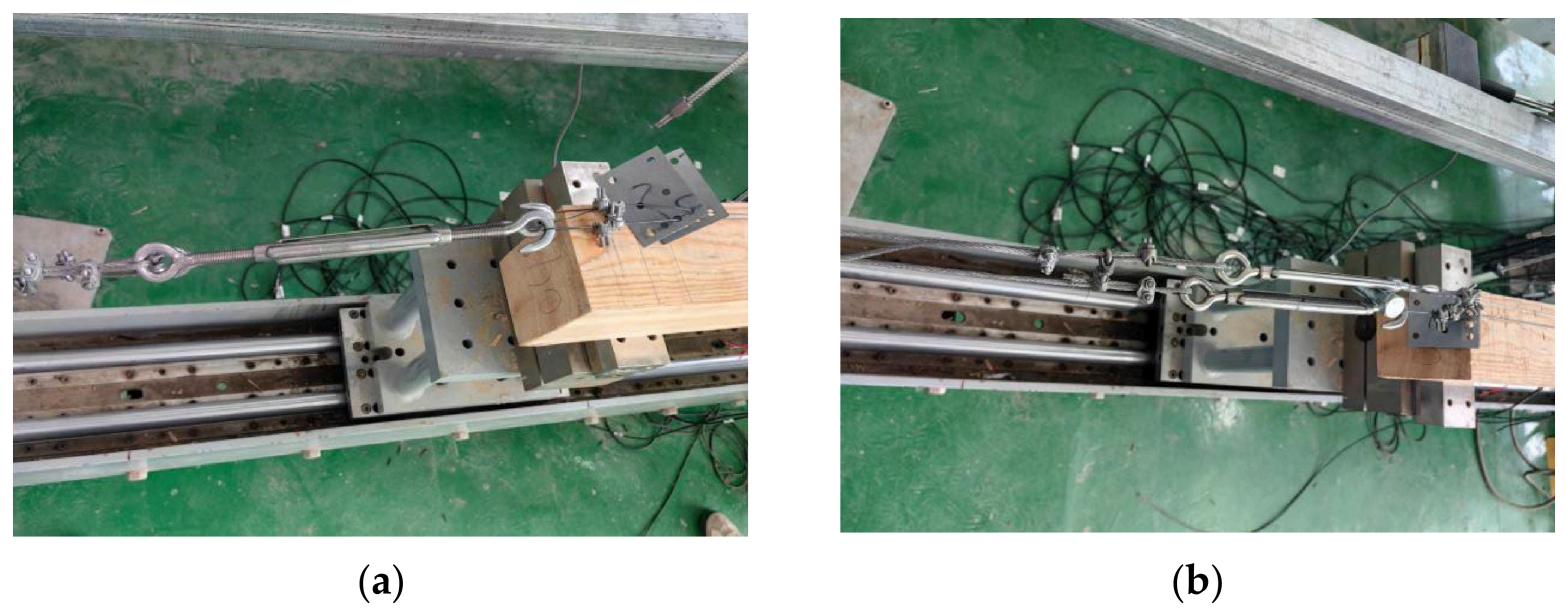
SMA wire tensioning process. (**a**) The SMA wire has not been tensioned. (**b**) The initial tensioning of the SMA strands has been completed.

When reinforcing test specimens L3-1, L4-1 and L5-1, the custom-made stainless steel plates were first secured on the left and right sides of the beam respectively. Then, in accordance with the test protocol, the prepared SMA wire was threaded through the wire-passing holes in the right-hand stainless steel plate and the end was secured with a clip. Finally, the SMA wire was allowed to extend naturally through the custom-made stainless steel plate on the left side of the beam and was secured with a clip. When reinforcing specimens L6-1, L7-1, L8-1, a new type of SMA wire tensioning device was employed. First, the SMA wire tensioning device was installed, and the SMA wire was secured on the right-hand side in accordance with the reinforcement method described above. A specific length of SMA wire was precisely measured in accordance with the test protocol, threaded through the unfixed threading hole in the left-hand stainless steel plate, and secured with a clip. At this stage, the stainless steel plate remained unfixed, as shown in Figure 12. The steel rods at both ends were secured to the loading platform frame. The steel wire rope with a hook at one end was wrapped around the right-hand steel rod, with the hook passed through the end of the SMA wire. The steel wire rope with a bolt tensioner at one end was wrapped around the left-hand steel rod, with the bolt tensioner end passed through the end of the SMA wire. The SMA wire tensioning device was then fully installed. Subsequently, in accordance with the test protocol, the bolt tensioner was rotated to control the degree of tension in the SMA wire, thereby completing the tensioning process.

It should be noted that the tensioning frame (shown in Figure 11) is used solely to apply prestress to the SMA wires prior to the bending test. Once the target prestress has been achieved, the tensioning bolts are locked in place, and the entire tensioning frame is removed from the loading platform before the four-point bending test commences. The prestress is maintained solely by the mechanical anchoring system (stainless steel plates and clamps at both ends of the timber beam, as shown in Figure 10), thereby creating a self-balancing prestressing system within the cross-section of the timber beam. This ensures that the SMA wire functions as an internal tensile reinforcement rather than an external loading device during the bending test.

**Figure 13:**
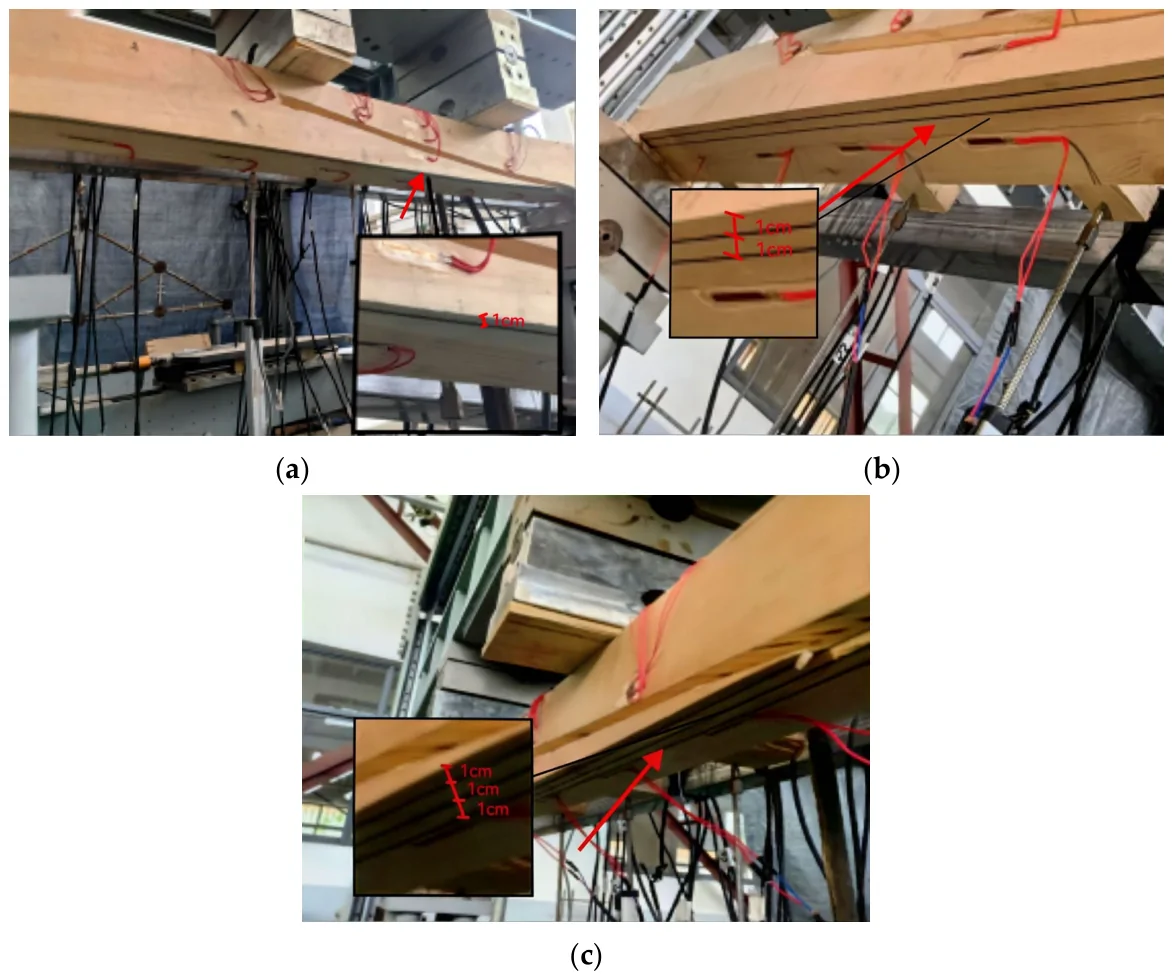
Location of SMA-reinforced timber beams. (**a**) 1 SMA wire. (**b**) 2 SMA wires. (**c**) 3 SMA wires.

To clearly illustrate the specific arrangement of the SMA wires on the bottom of the beam, Figure 13 shows schematic cross-sectional views of test specimens reinforced with 1, 2 and 3 SMA wires, respectively. All SMA wires are positioned in the tension zone at the bottom of the beam and are distributed at equal intervals across the beam width. Of these, the three wires are evenly distributed across the beam width. The SMA wires pass through pre-drilled channels in the bottom of the beam and are anchored at both ends to the beam ends using custom-made stainless steel plates and clamps. For the prestressed specimens (L6-1 to L8-1), prestress was applied using a bolt tensioner after the wires had been installed; once tensioning was complete, the tensioning frame was removed, and the prestress was maintained by the end anchorage system. Figure 13 illustrates the relative positions and spacing of the SMA strands, as well as their spatial relationship with the crack surface.

#### 2.4.3. Loading System

**Figure 14:**
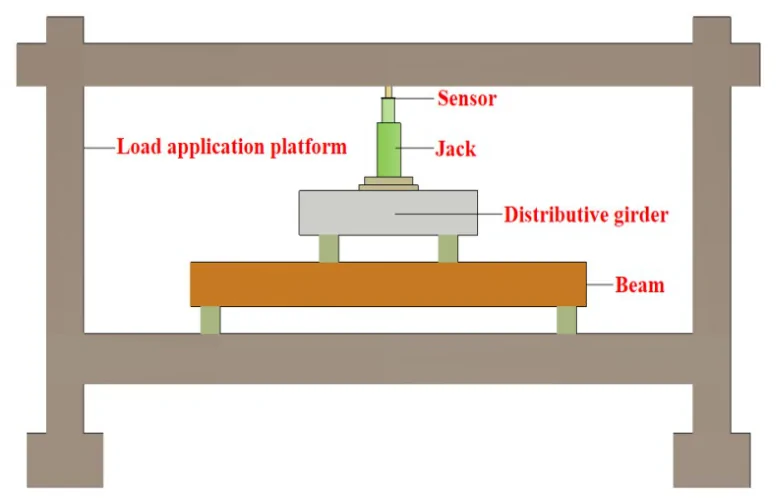
Schematic diagram of the four-point bending loading device.

This test involves a four-point bending test on a timber beam [28]. A hydraulic jack is used to apply a vertical load to the beam; the jack is connected to a pressure transducer, a steel distributive girder is used to transfer the hydraulic jack load to two loading points located at the one-third span positions, creating a pure bending zone between them, and the load values are recorded using a strain data logger. A schematic diagram of the loading apparatus is shown in Figure 14.

With reference to [29,30], the loading procedure is divided into two stages: the pre-loading test and the main loading test. Prior to the main loading test, a pre-loading test was conducted on the timber beam, applying 10% of the beam’s ultimate load-bearing capacity to verify that the strain acquisition system was functioning correctly and that the readings from the displacement transducer and strain gauges were reasonable. Once the system had been successfully calibrated, the main loading test commenced. The main loading was carried out using a step-by-step loading method. During the loading process, the failure behaviour of the timber beam was observed and recorded. Data were manually collected once the readings had stabilised after each loading step, and loading was continued until the timber beam failed.

#### 2.4.4. Layout of Measurement Points

**Figure 15:**
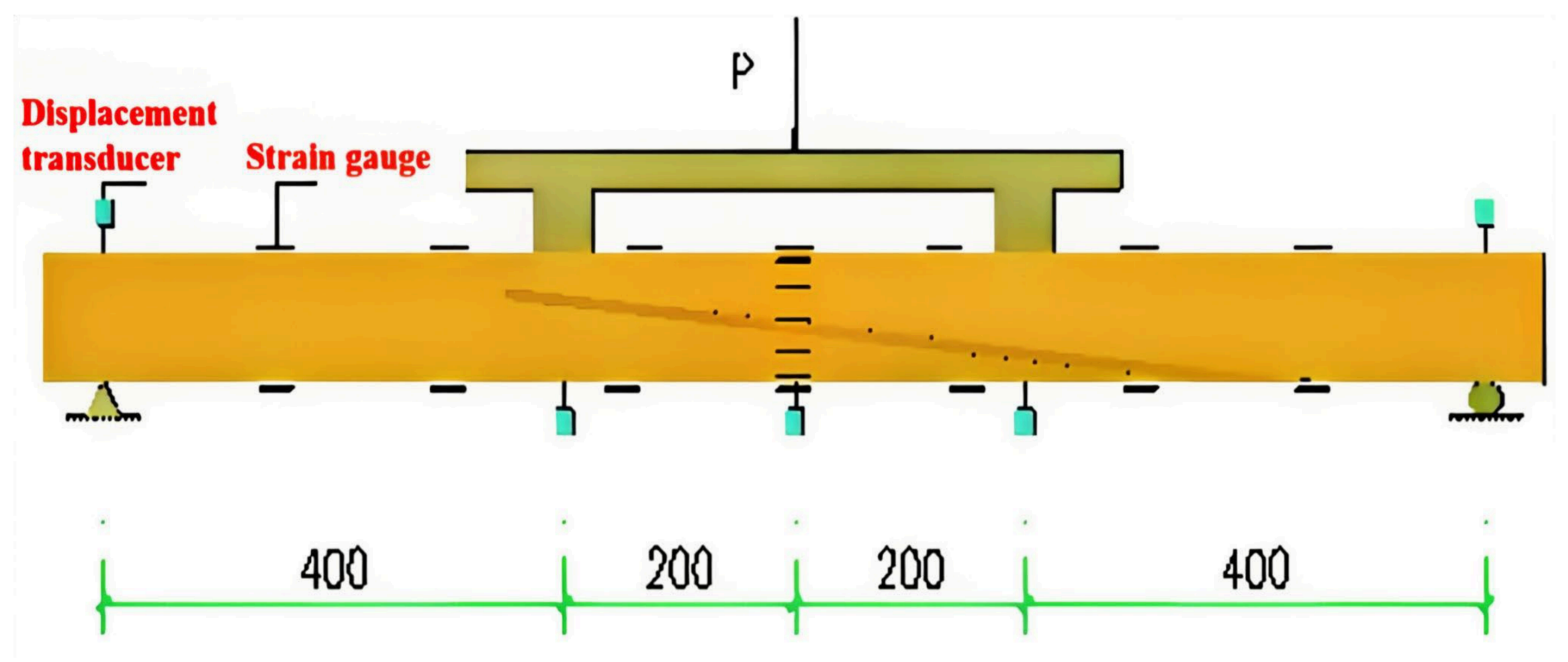
Schematic diagram of the measurement point layout (four-point bending configuration with loading points at the one-third span positions).

A total of 28 strain gauges were installed for this test, with seven each placed in the compression and tension zones of the timber beam at 15 mm intervals. Due to the influence of the shims in the compression zone of the timber beam, the spacing between the strain gauges varied slightly. A total of five strain gauges were uniformly arranged at the mid-span of the timber beam, with the remaining strain gauges forming strain rosettes, positioned at the ends of the cracks and on the sides of the cracks. A total of five displacement transducers were installed in this test, located at the ends of the timber beam, the mid-span, and the left and right third points. The arrangement of the measurement points is shown in Figure 15.

To monitor the actual tensile force of the SMA wires during the bending test, two high-precision strain gauges (model BX120-3AA is supplied by Yiyang Yingzhen Testing Technology Co., Ltd., Beijing, China, sensitivity coefficient 2.08 ± 1%) were affixed to the surface of each SMA wire at the mid-span position (where the maximum tensile strain was expected). The strain gauges were installed in accordance with the manufacturer’s recommended procedure, which included surface cleaning with acetone, application of cyanoacrylate adhesive and the application of a protective coating. The strain readings from the SMA wires and the strain data from the timber beams were recorded simultaneously using the same data acquisition system (DH3816N static strain tester). The tensile force on each SMA wire was calculated using the formula F = E·ε·A, where E = 83 GPa (austenitic modulus of elasticity; see Table 1), ε is the measured strain, and A = 1.131 mm^2^ is the cross-sectional area of a single wire.

The strain readings from the SMA wire strain gauges were recorded using the same static strain data acquisition system (DH3816N, supplied by Donghua Testing Technology Co., Ltd., Taizhou, Jiangsu Province, China) as that used for the timber strain gauges, with a sampling frequency of 1 Hz. Each strain gauge was calibrated by the manufacturer with a sensitivity coefficient of 2.08 ± 1%. Prior to the application of prestress, the initial readings of all SMA wire strain gauges were recorded and set as the zero reference. All subsequent strain values were calculated as the difference between the current reading and this initial reference value, thereby eliminating any strain effects caused by initial wire relaxation or installation. For specimens equipped with two SMA wires (L4-1, L6-1, L7-1, L8-1), the strain values for each wire were measured separately; the tensile force for each wire was calculated individually, and the total force was obtained by summing these values. No temperature compensation was applied during the tests, as all measurements were carried out under stable laboratory conditions (room temperature maintained at 23 ± 2 °C). The strain values presented in this study are engineering strains, calculated directly from the strain gauge readings without correction for calibration coefficients.

### 2.5. Description of Test Phenomena

#### 2.5.1. Test Observations for the Undamaged Timber Beam

**Figure 16:**
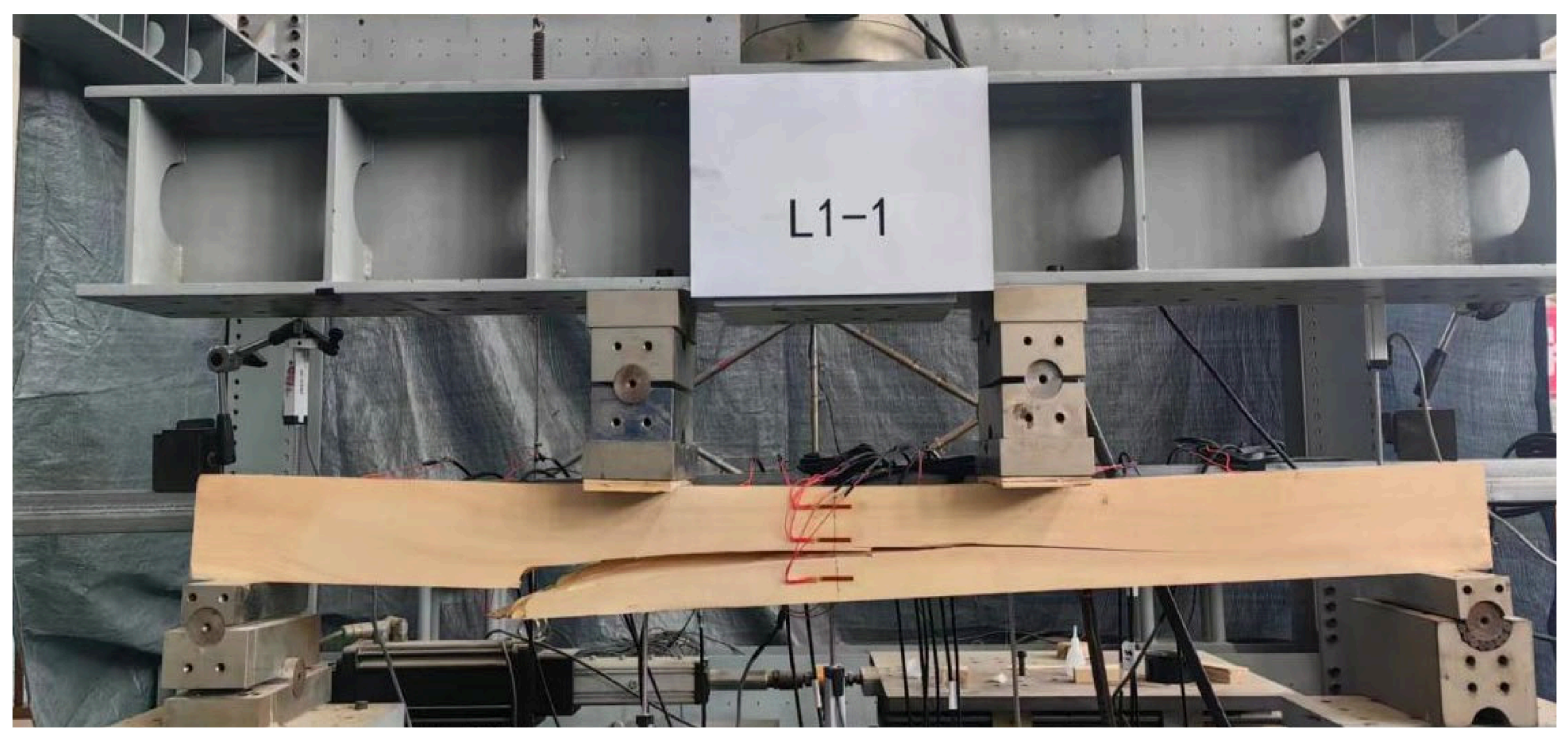
Failure diagram of the intact timber beam specimen L1-1.

The intact timber beam specimen L1-1 showed no obvious cracks or large knots on the underside. During the initial loading phase, the timber beam exhibited no significant changes; as the load increased, the beam exhibited slight deflection, and a faint sound of wood fibre breaking was heard at the left loading point. When the load P reached 11.1 kN, the wood fibres at the left loading point on the bottom of the beam suddenly broke, ejecting a small amount of wood shavings; a fine crack appeared 8.3 cm from the left loading point, extending from the bottom of the beam towards the mid-span at a 40° angle, with a length of 20.2 cm; during continued loading, when the load P reached 12.8 kN, a distinct cracking sound was heard from the timber beam, and the crack at the left loading point began to propagate; when the load P reached 14.4 kN, a continuous, dense series of wood fibre breaking sounds occurred at the left loading point, and the crack showed significant propagation; as the load was further increased, at a load of P = 18 kN, accompanied by a loud bang, the crack rapidly extended to the right loading point, ejecting a large amount of wood shavings, and the specimen failed. The failure mode is shown in Figure 16.

#### 2.5.2. Test Observations for the Cracked Wooden Beam

**Figure 17:**
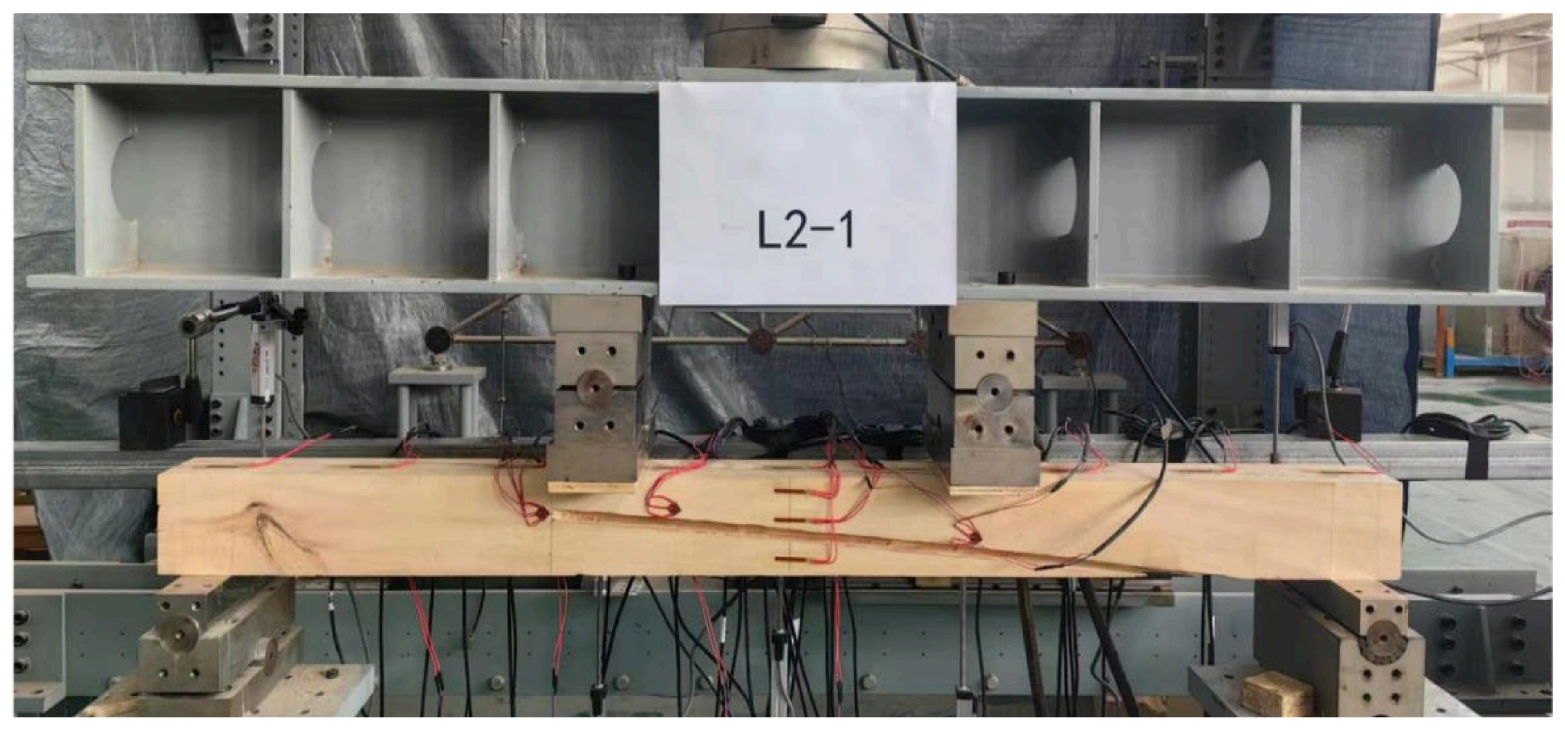
Schematic diagram of the cracked state of timber beam L2-1. (Key geometric parameters: horizontal projected length on the bottom surface = 190 mm; depth = 20 mm; width = 10 mm; inclination angle relative to the beam bottom surface = 6°; and notch starts at the bottom centre of the right shear-bending zone and extends towards the mid-span).

The cracked timber beam specimen L2-1 had a knot on its left side, with no obvious cracks on the bottom of the beam. A crack was artificially introduced to simulate a cracked condition; this crack extended from the centre point at the bottom of the right-hand shear-bending section towards the mid-span at a 6° angle, with a horizontal projected length of 190 mm, a width of 10 mm and a depth of 20 mm. The cracked condition is shown in Figure 17.

**Figure 18:**
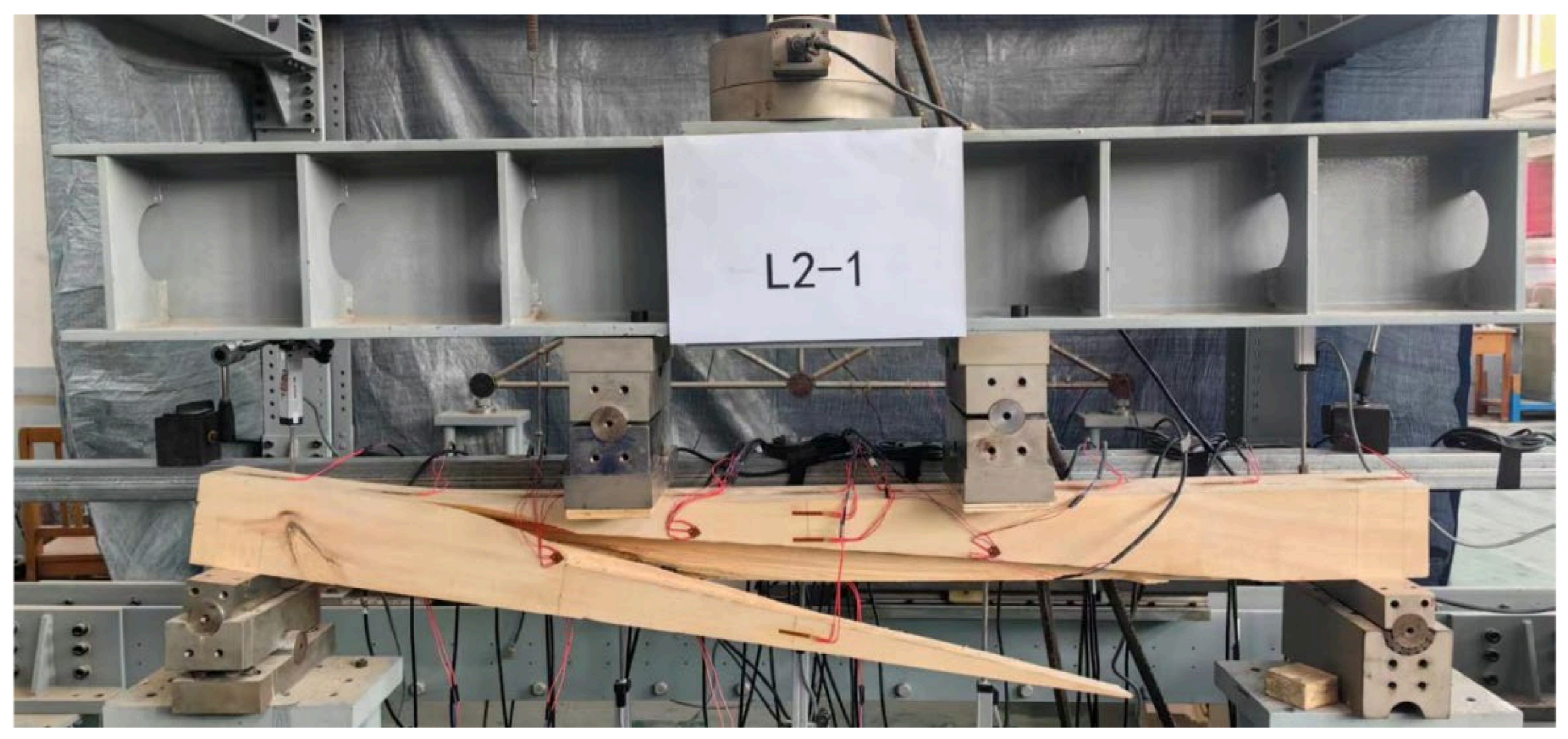
Failure diagram of cracked timber beam L2-1.

During the initial loading phase, the timber beam showed no significant changes and remained in the elastic stage; as loading continued, sporadic cracking sounds from breaking wood fibres were heard at the right loading point when the load P reached 6.6 kN; when the load reached P = 8.6 kN, a crisp sound of wood fibre breaking was heard at the end of the crack in the right-hand shear-bending section, and a fine crack appeared along the direction of the artificially created damage; when the load reached P = 10.8 kN, a loud bang was heard at the end of the crack on the right-hand side of the beam, and the fine crack rapidly extended towards the pure bending section; during continued loading, at P = 11.4 kN, continuous sounds of wood fibre breaking were heard throughout the beam; the artificially created crack extended from its left end, whilst the fine cracks on the right side of the beam continued to expand; at P = 12.1 kN, accompanied by a loud bang, the timber beam fractured along the crack, lost its load-bearing capacity, and the specimen failed. The failure mode is shown in Figure 18.

#### 2.5.3. Test Observations for Reinforced Timber Beams

(1) Reinforced timber beam L3-1

**Figure 19:**
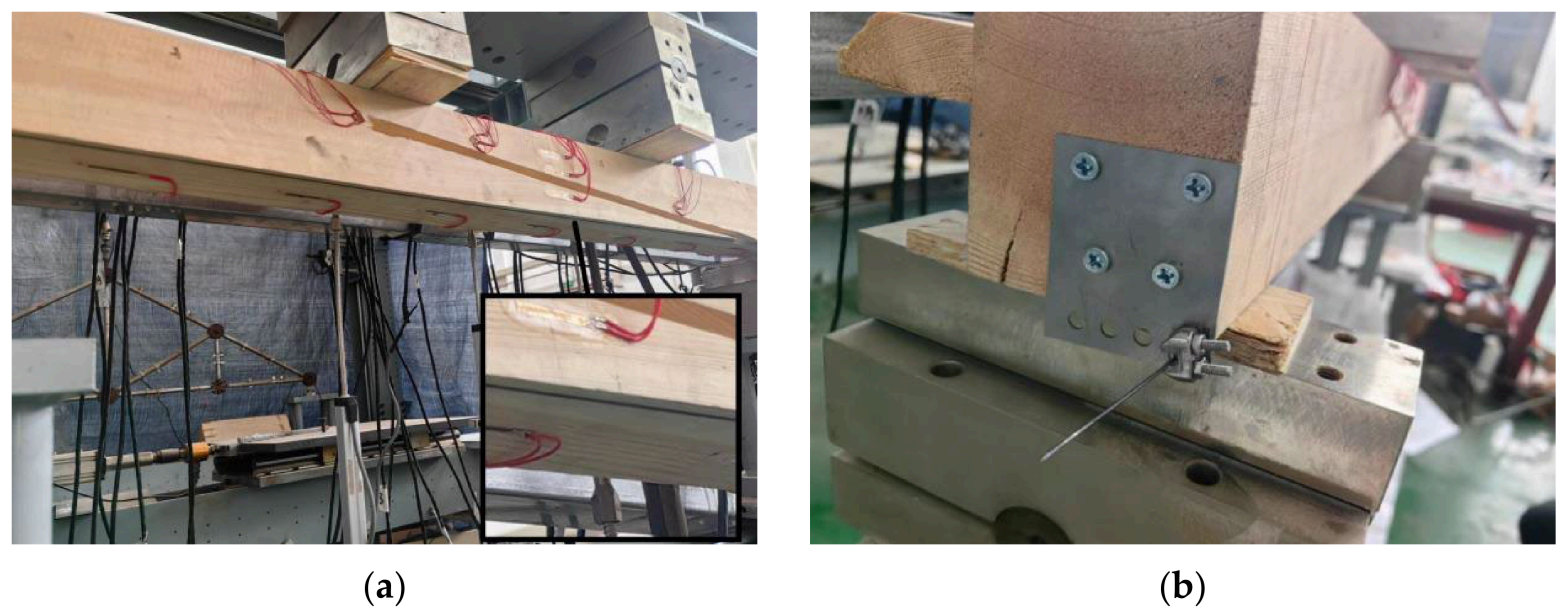
Schematic diagram of the reinforcement method for timber beam L3-1. (**a**) Schematic diagram of the SMA wire position. (**b**) Schematic diagram of SMA wire fixation.

The reinforced timber beam specimen L3-1 had no obvious knots or cracks in the beam body. A crack identical to that in L2-1 was created, and a shape memory alloy (SMA) wire was installed 1 cm from the bottom of the beam to reinforce it, positioned directly beneath the crack. Both ends of the shape memory alloy wire were secured using custom-made stainless steel plates and locked in place with clips to prevent the wire from slipping during the test, thereby ensuring its performance was fully utilised. The reinforcement method is shown in Figure 19. The above methods are referenced in [13,14].

**Figure 20:**
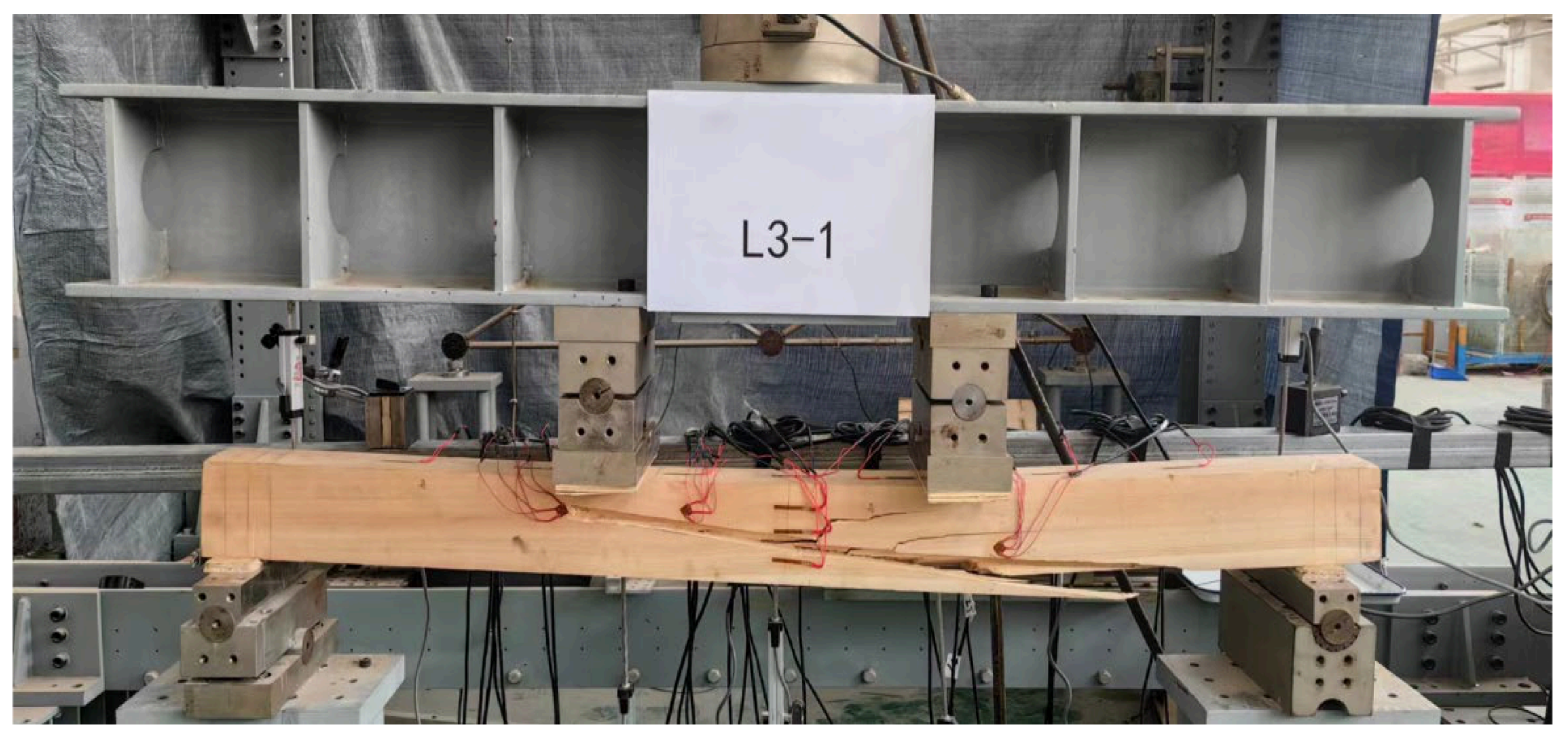
Failure diagram of reinforced timber beam L3-1.

During the initial loading phase, the timber beam remained in the elastic stage, and the SMA wire gradually bore tensile forces; when the load P reached 6.8 kN, sporadic sounds of wood fibre breaking were heard at the crack tip; with continued loading, at a load of P = 9.4 kN, a crisp sound of wood fibre breaking was heard at the right end of the crack, accompanied by the ejection of a small amount of wood shavings; with continued loading, at a load of P = 11.2 kN, sporadic cracking sounds were heard throughout the beam; at a load of 12.7 kN, rapid sounds of wood fibre breaking were heard throughout the beam; finally, at a load of P = 13.5 kN, the timber beam emitted a loud crack and fractured along the damaged crack, losing its load-bearing capacity. At this point, the SMA wire was in a tensile state and had not become embedded in the timber beam. The failure mode of the timber beam is shown in Figure 20.

(2) Reinforced timber beam L4-1

**Figure 21:**
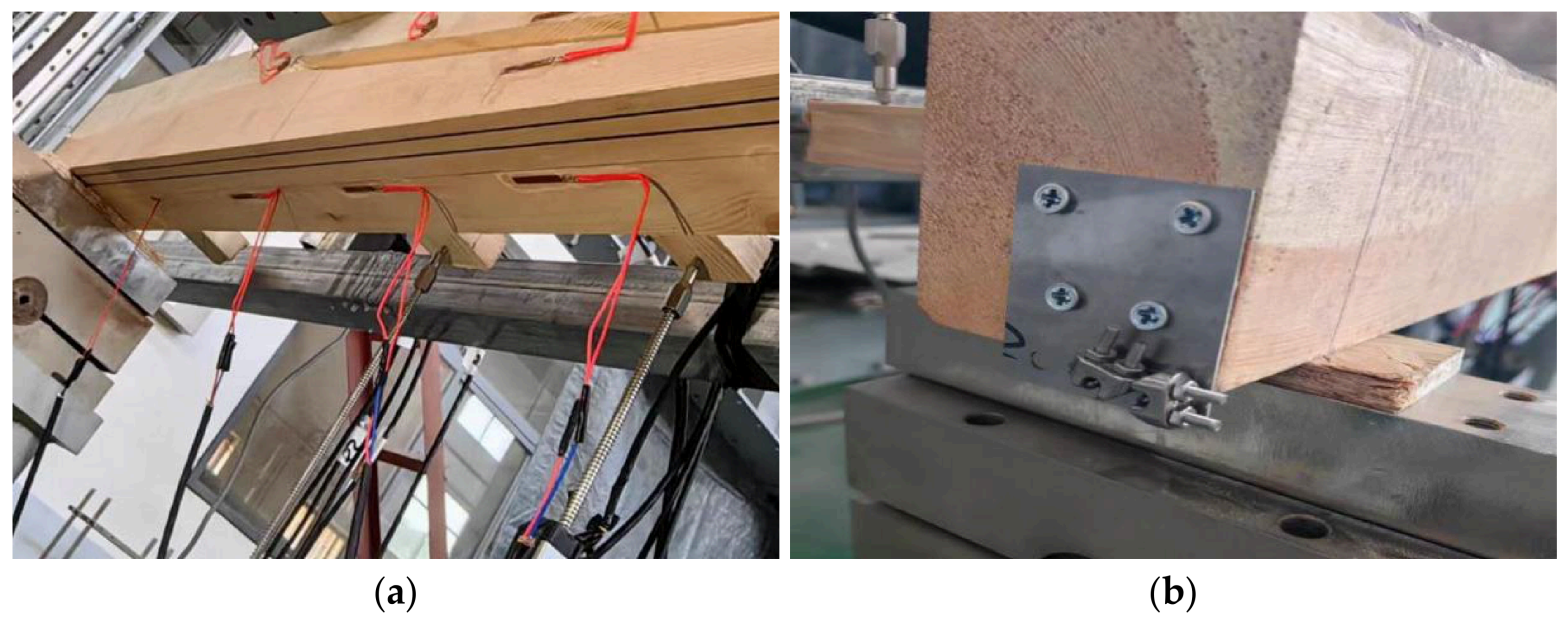
Schematic diagram of the reinforcement method for timber beam L4-1. (**a**) Schematic diagram of SMA wire positions. (**b**) Schematic diagram of SMA wire fixation.

The reinforced timber beam specimen L4-1 contained a small knot in the mid-span compression zone; a crack pattern identical to that of L2-1 was artificially created. Two SMA wires were installed at the bottom of the specimen, positioned 1 cm and 2 cm from the beam edge at the base of the crack, respectively. Both ends of the SMA wires were secured using custom-made stainless steel plates and clips. The reinforcement method is shown in Figure 21.

**Figure 22:**
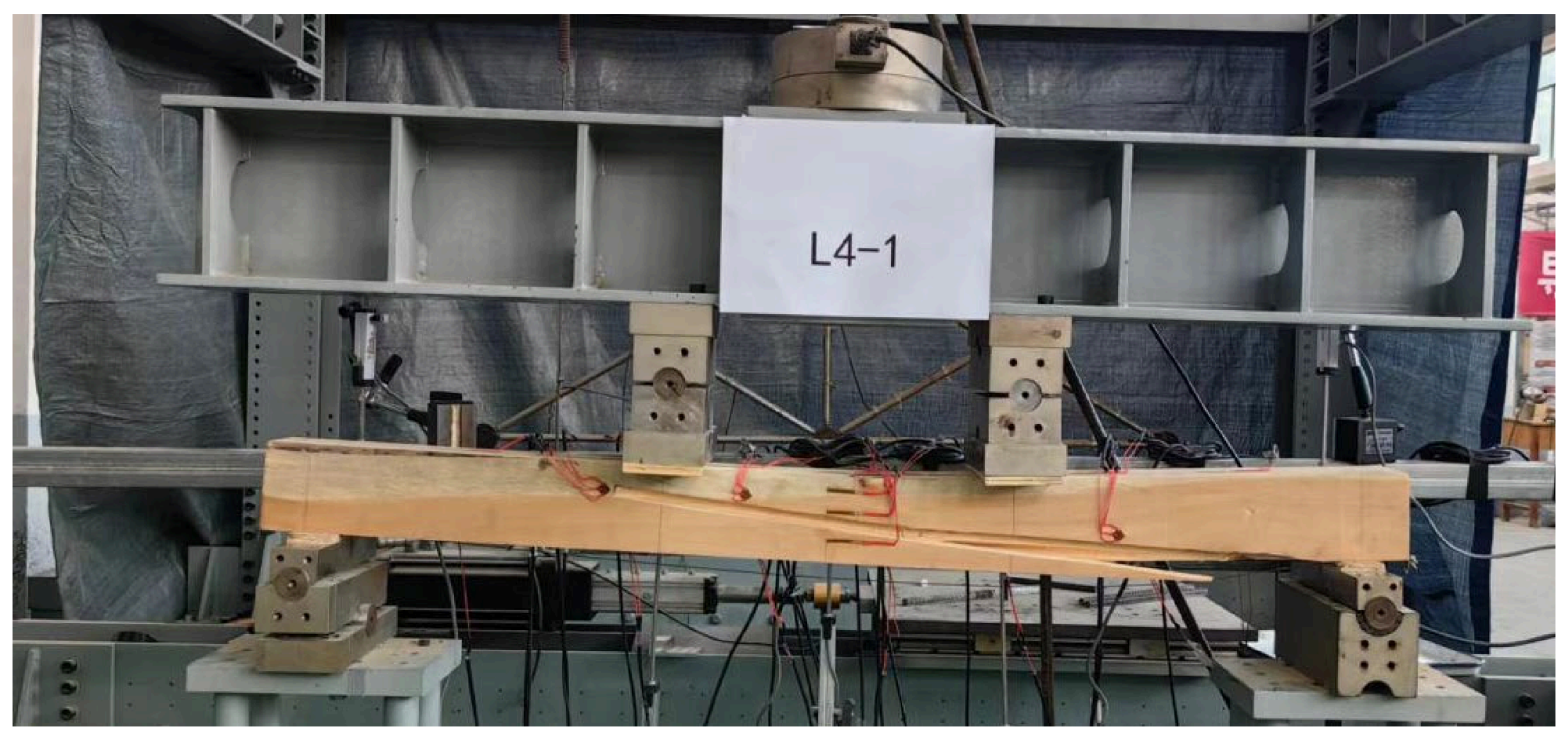
Failure diagram of reinforced timber beam L4-1.

During the initial loading phase, the timber beam gradually came under stress without any noticeable phenomena; as loading continued, when the load P reached 8.1 kN, a faint cracking sound was heard from the timber beam, and the SMA wires remained taut under continuous stress; as loading continued, when the load P reached 10.2 kN, the timber fibres on the left side of the beam tore, producing a sound of fibre fracture; at a load of P = 12.4 kN, the cracking sounds from the beam body gradually increased, and the SMA wires were under tension; at the ultimate load of P = 13.9 kN, the fibres at the crack in the timber beam broke, and the beam failed with a loud bang; the SMA wires remained in place at the bottom of the timber beam and continued to function, preventing the beam from snapping suddenly. The failure mode of the specimen is shown in Figure 22.

(3) Reinforced timber beam L5-1

**Figure 23:**
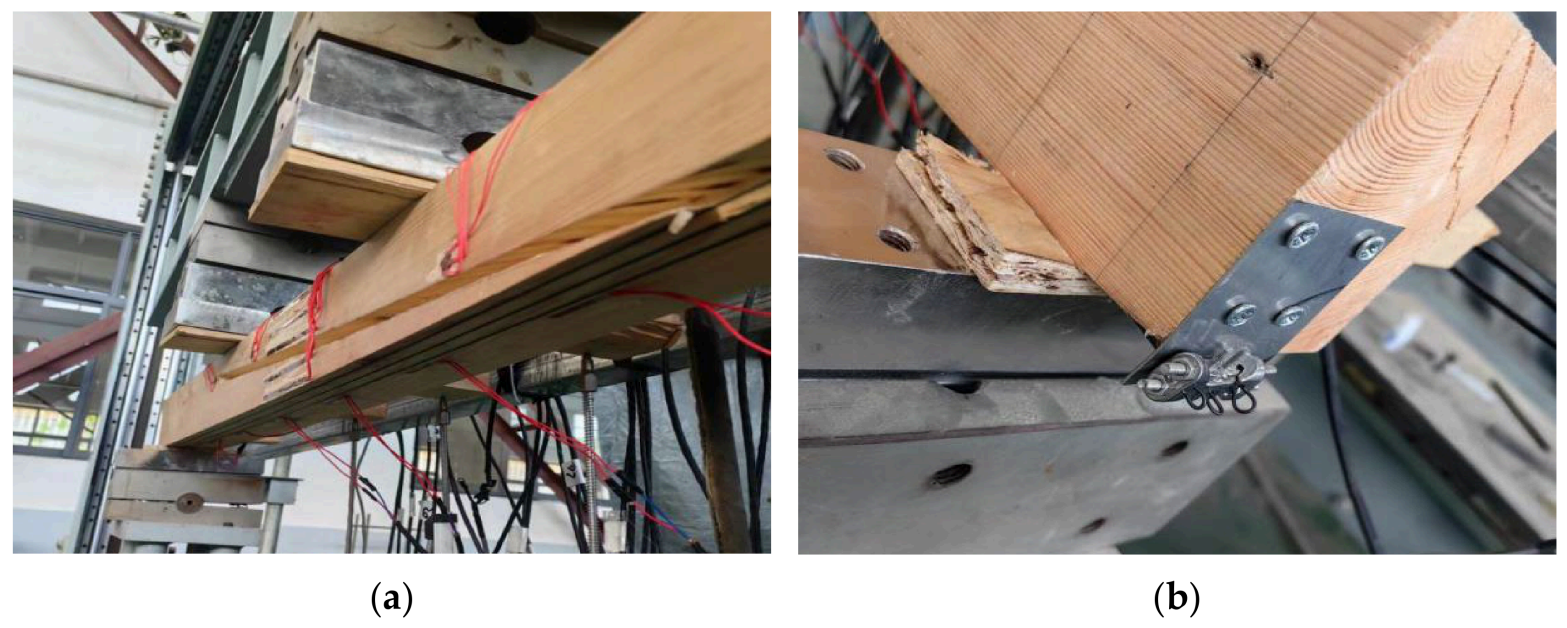
Schematic diagram of the reinforcement method for timber beam L5-1. (**a**) Schematic diagram of SMA wire positions. (**b**) Schematic diagram of SMA wire fixation.

The reinforced timber beam L5-1 specimen exhibited a small knot in the compression zone, whilst the tension zone at the bottom showed no significant damage. Three shape memory alloy wires were installed at the bottom of this specimen and secured to the sides of the beam using custom-made stainless steel plates; the reinforcement method is shown in Figure 23. The SMA wires were positioned 1 cm, 2 cm and 3 cm from the beam side at the bottom of the beam, respectively, and no prestress was applied prior to loading.

**Figure 24:**
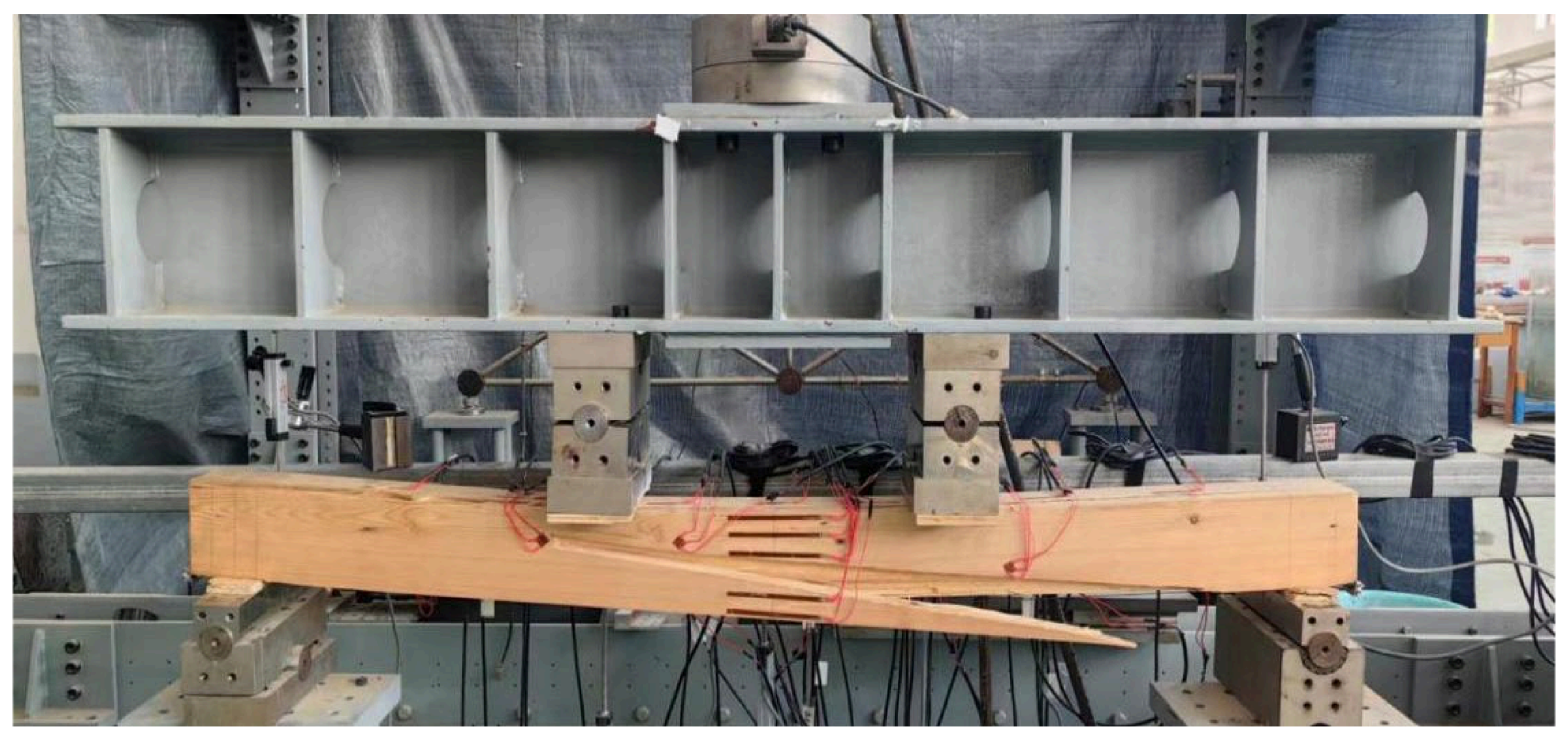
Failure of reinforced timber beam L5-1.

During the initial loading phase, the timber beam remained in the elastic stage, with no significant changes to the beam body, whilst the SMA wires remained taut; when the load P reached 5.8 kN, sporadic sounds were heard from the beam body; as loading continued, at a load of P = 8.4 kN, a small crack appeared at the end of the crack on the right side of the timber beam, accompanied by the sound of wood fibres breaking; at a load of P = 11.9 kN, the small crack in the timber beam gradually extended, causing wood shavings to splinter out; with continued loading, at a load of P = 13.4 kN, continuous sounds of wood fibres snapping were heard from the beam, accompanied by rapid ‘cracking’ noises; as the load increased to 14.5 kN, a loud bang was heard, followed by the failure shown in Figure 24, with the timber beam fracturing along the crack.

(4) Reinforced timber beam L6-1

**Figure 25:**
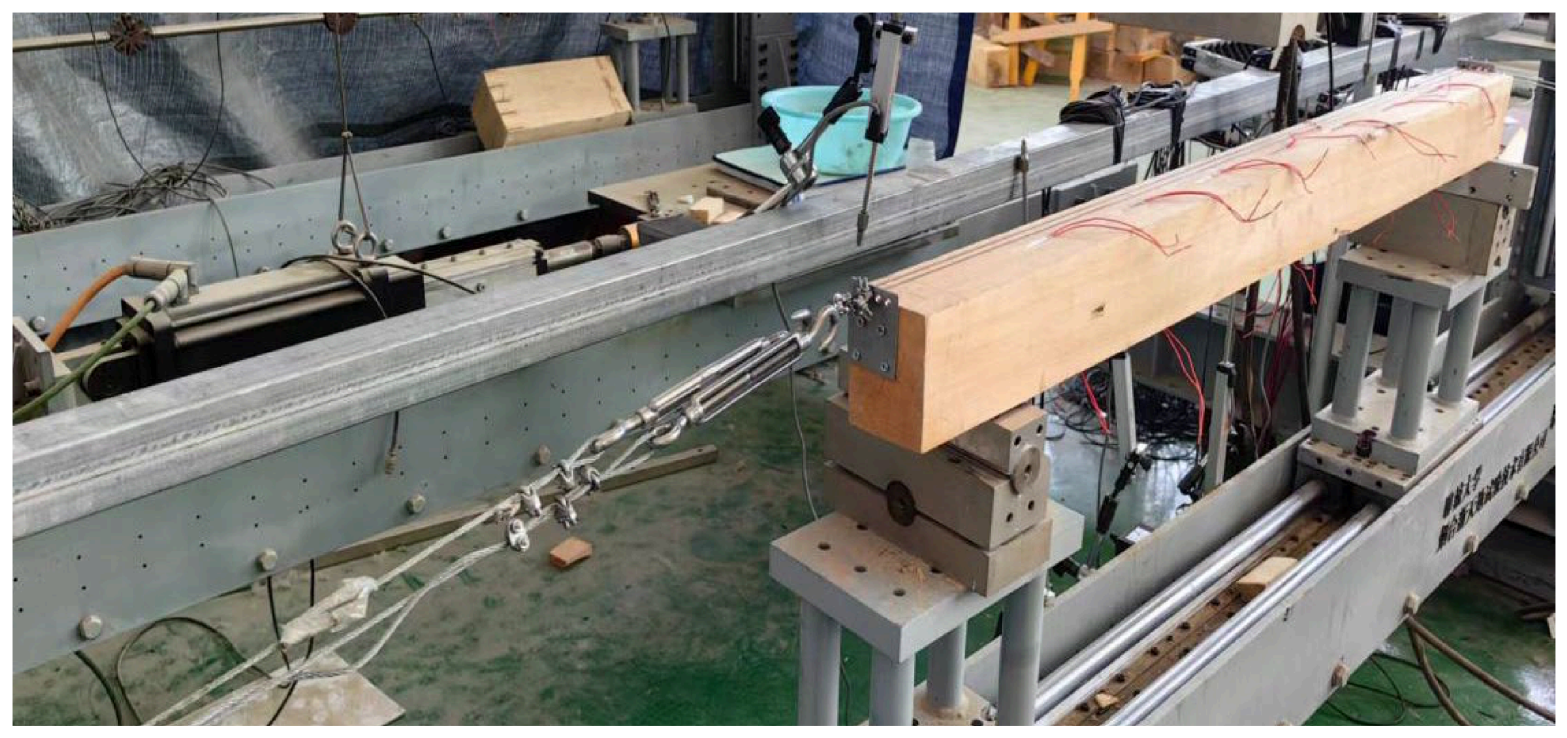
Schematic diagram of the reinforcement method for timber beam L6-1.

The reinforced timber beam specimen L6-1 exhibited a small knot above the right-hand end of the crack, with no other obvious damage. Two SMA wires were arranged at the bottom of this specimen, in the same positions as in specimen L4-1. The initial length of each SMA wire was 128.7 cm. Using the SMA wire tensioning device, the wire was elongated by 1.3 cm (i.e., to a total length of 130.0 cm); Figure 25 shows the SMA wire in its fully tensioned state.

**Figure 26:**
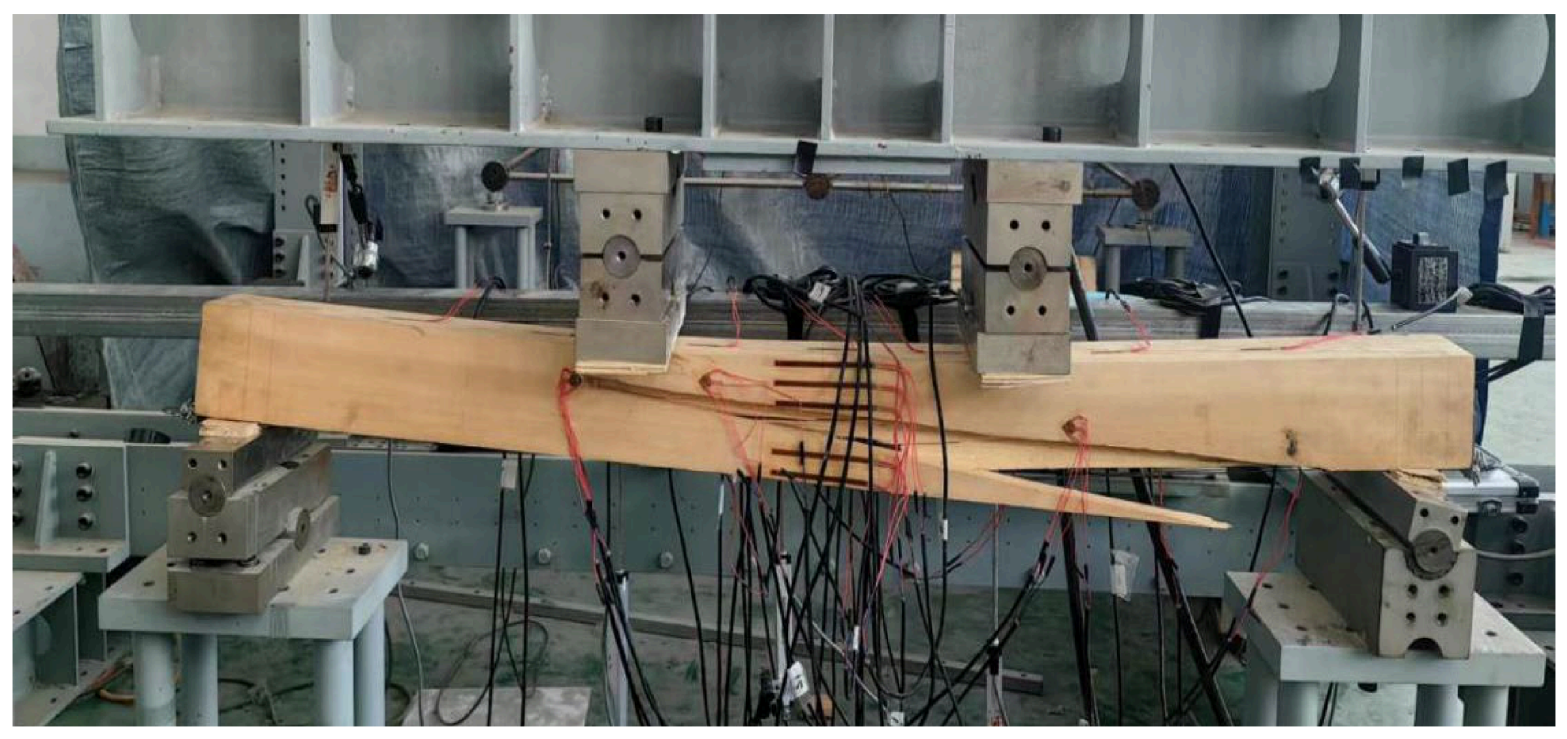
Failure of reinforced timber beam L6-1.

At the start of loading, slight bending of the timber beam was observed, with no distinct sound of wood fibre fracture; as loading continued, when the load P reached 7.1 kN, a faint sound was emitted due to the close compression between the beam body and the supports; as the load was further increased, when P reached 9.4 kN, a crackling sound emerged from the entire beam body, indicating the onset of wood fibre fracture within the timber beam; as loading continued, when the load reached P = 12.1 kN, a continuous cracking sound emanated from the entire beam, gradually becoming more rapid; as loading continued further, when the load reached P = 15.3 kN, a loud bang occurred, the wood fibres at the base of the crack were torn apart, and the beam fractured along the crack direction as shown in Figure 26, resulting in the failure of the specimen.

(5) Reinforced timber beam L7-1

**Figure 27:**
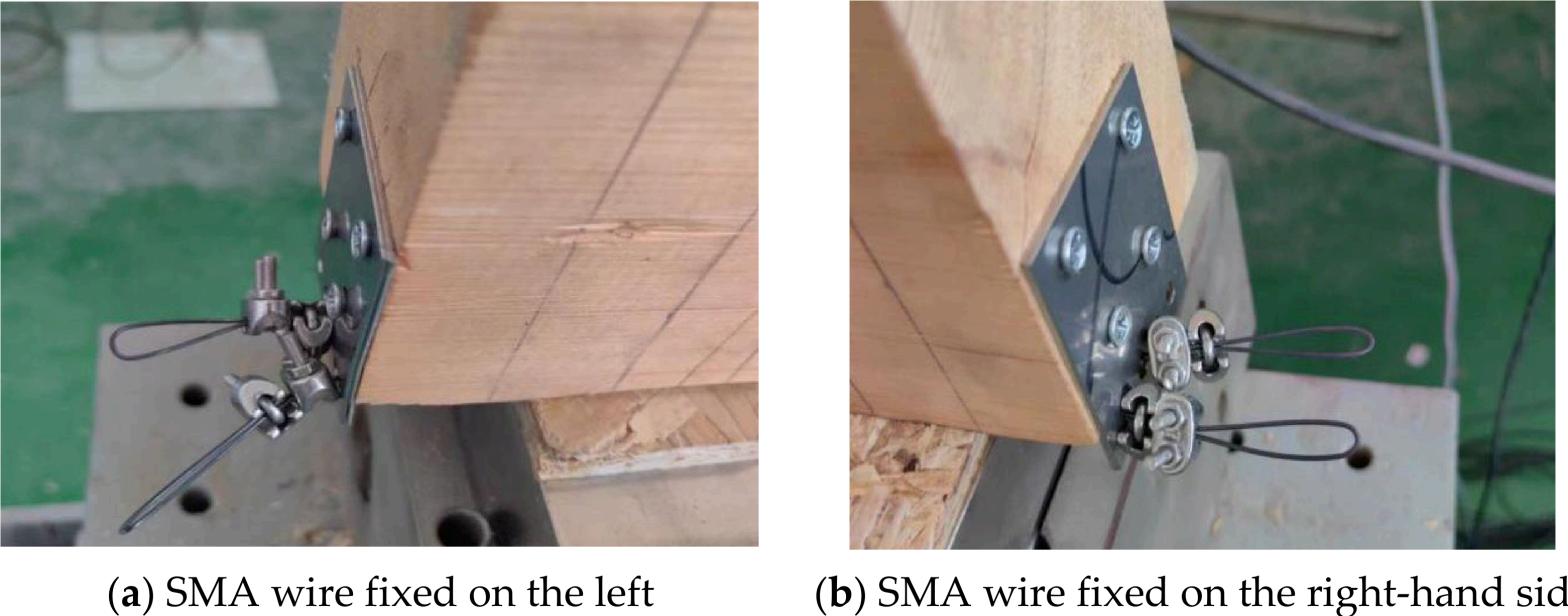
Schematic diagram of the SMA wire fixing method for the reinforced timber beam L7-1.

Apart from artificially induced damage, the body of the reinforced timber beam L7-1 specimen showed no obvious cracking. Two SMA wires were positioned at the bottom of the timber beam in the same locations as in specimen L4-1, with an initial length of 127.4 cm. Each shape memory alloy wire was fixed on the right-hand side and secured to the loading platform via a hook; tension was applied using two bolt tensioners on the left-hand side, with a tensioning length of 2.6 cm. Before the tensioning was fully completed, the shape memory alloy wires were secured at the ends of the timber beam using two clamps each, as shown in Figure 27.

**Figure 28:**
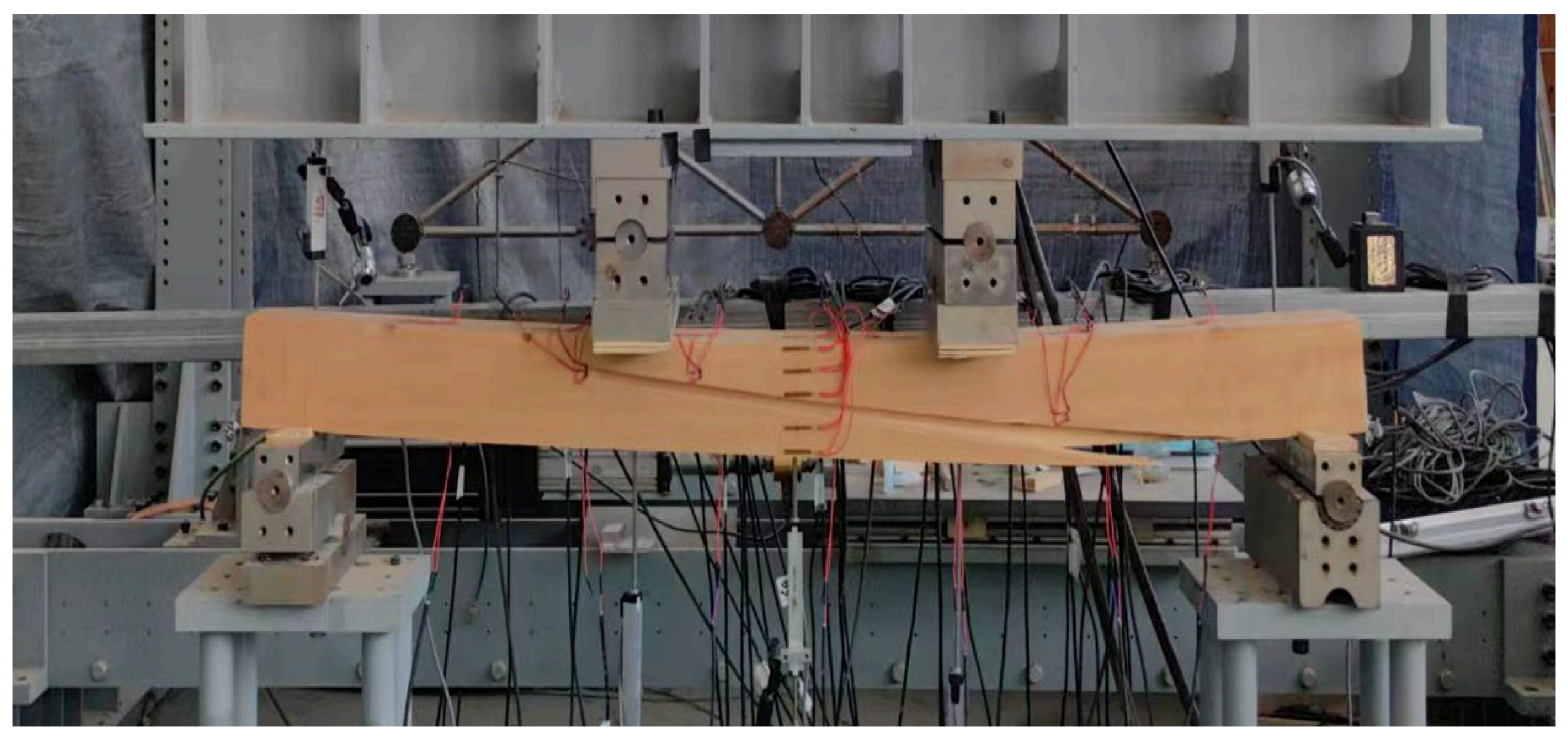
Failure of reinforced timber beam L7-1.

During the initial loading phase, no obvious phenomena were observed in the timber beam. Upon continuous loading until the load reached P = 9.1 kN, sounds of timber fibres being compressed were heard from both the upper loading point and the lower support; as the load was further increased, a distinct sound of timber fibres breaking at the crack was heard when the load reached P = 10.4 kN, although no obvious cracks had formed on the surface of the beam; as loading continued to P = 13.2 kN, accompanied by sporadic sounds of wood fibres snapping, increased resistance was distinctly felt during loading, and the timber beam showed clear signs of bending; when the load was increased to P = 16.1 kN, the timber beam suddenly fractured with a loud bang, and the specimen failed, as shown in Figure 28.

(6) Reinforced timber beam L8-1

Apart from a small knot at the edge of the crack near the right-hand loading point, the reinforced timber beam L8-1 specimen was generally in good condition. A damage condition identical to that of the cracked timber beam L2-1 was artificially created by placing two SMA wires on the bottom of the beam, with a prestress of 3%, in the same positions as those on the reinforced timber beam L4-1. The initial length of the SMA wires used for this specimen was 126.1 cm, and they were tensioned using a tensioning device by 3.9 cm.

**Figure 29:**
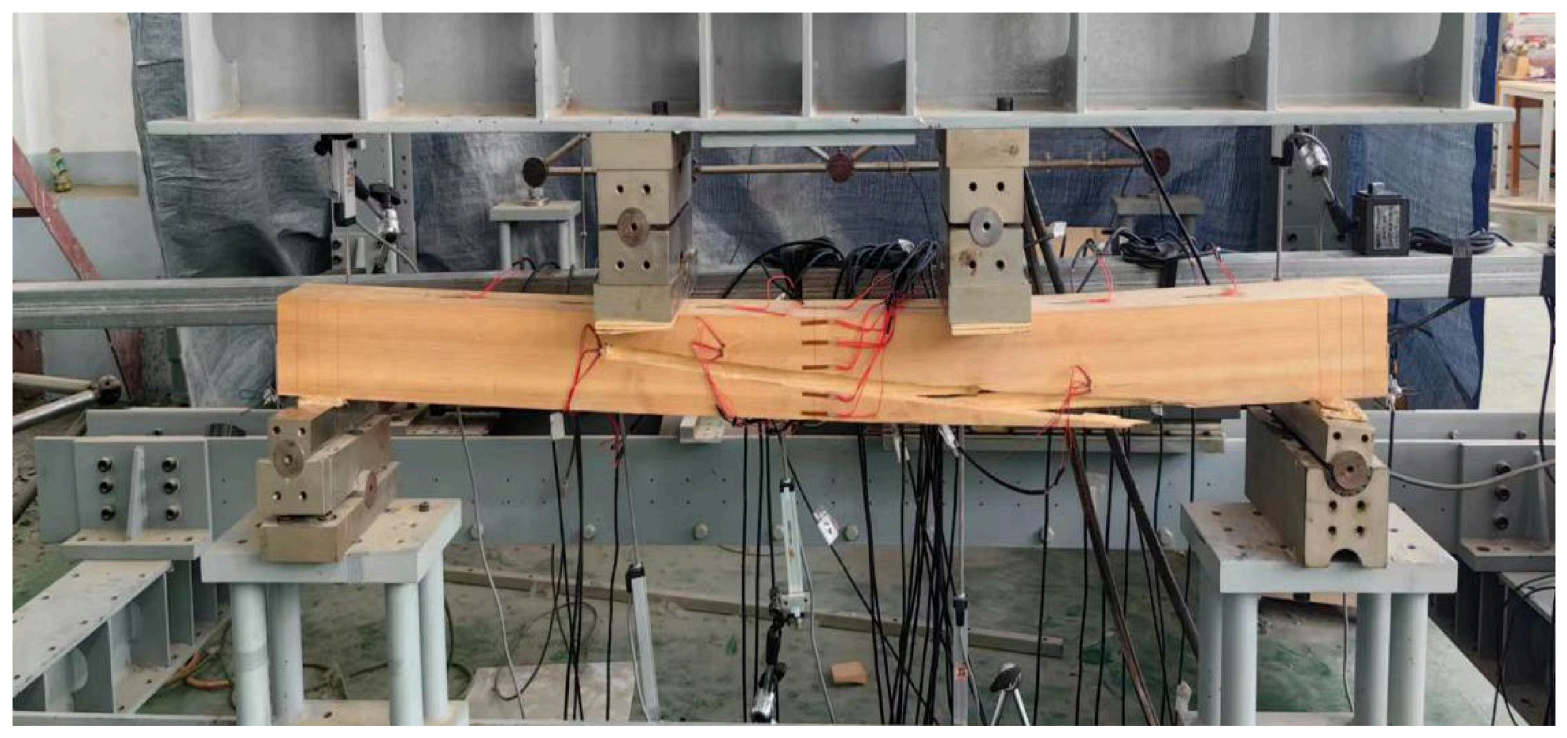
Failure of reinforced timber beam L8-1.

During the initial loading phase, the timber beam showed no significant changes and remained in the elastic stage; as the load was continuously increased, when the load P reached 11.094 kN, a faint sound of wood fibre breaking was heard at the initial knot, accompanied by a crackling sound at the crack tip; as the load was further increased, a noticeable increase in resistance was felt when applying the load via the jack; when the load reached P = 15.5 kN, a distinct ‘crack’ was heard from the initial knot of the timber beam, a crack appeared, a small amount of wood shavings were ejected, and sporadic cracking sounds were heard along the beam; as loading continued to P = 16.7 kN, accompanied by rapid sounds of wood fibre fracture along the beam, the crack at the knot extended, as shown in Figure 29. The timber beam lost its load-bearing capacity and the specimen failed.

To facilitate systematic comparison and analysis, the failure modes, key characteristics and corresponding stress behaviour of all test specimens are summarised in Table 18. This table covers intact beams, cracked beams and reinforced beams with varying numbers of SMA wires and prestressing levels, focusing on the crack initiation location, propagation path, ultimate failure mode and the role of the SMA wires during the failure process. A comparison reveals that the introduction of SMA wires, particularly when combined with increased prestressing levels, causes the failure mode to gradually shift from brittle tensile fracture to more ductile crushing in the compression zone, thereby significantly improving the load-bearing performance of the test specimens.

## 3. Results and Discussion

### 3.1. Ultimate Load-Bearing Capacity

During the loading process, the timber beam fractured distinctly along the damaged direction with a loud bang, and the load value collected by the strain acquisition instrument began to show a downward trend. At this point, loading on the timber beam was ceased, and the beam lost its bearing capacity. The ultimate bearing capacity results of the test members are presented in Table 19. It can be seen from the table that the ultimate bearing capacity of the damaged timber beam is significantly lower than that of the intact one; the ultimate bearing capacity of the timber beam reinforced with shape memory alloy (SMA) wires is notably higher than that of the damaged beam, and also shows an increase to varying degrees compared with the intact beam.

When no pre-strain was applied to the SMA wires and only the number of wires was varied, the ultimate bearing capacity of the timber beam showed an increasing trend with a greater number of wires: 13.5 kN (L3-1, 1 wire), 13.9 kN (L4-1, 2 wires) and 14.5 kN (L5-1, 3 wires). However, given the inherent variability of timber (typical coefficient of variation of 15–25% in bending strength) and the presence of knots in some specimens (e.g., a knot in the mid-span compression zone of L4-1), these differences (0.4–1.0 kN) are within the expected range of material scatter. Therefore, while the results suggest a positive trend, they do not provide statistically conclusive evidence that increasing the number of SMA wires improves the ultimate load-bearing capacity. The observed trend warrants further investigation with a larger number of replicate specimens.

When two SMA wires were installed at the bottom of the timber beam and only the pre-strain of the wires was adjusted, the ultimate bearing capacity increased more obviously with a higher pre-strain level. The SMA wires used for Specimen L6-1 had a pre-strain of 1%, leading to a significant 26.45% increase in ultimate bearing capacity compared with the damaged beam, and a higher ultimate bearing capacity than Specimen L4-1 with the same number of reinforcement wires and reinforcement position; the SMA wires for Specimen L7-1 had a pre-strain of 2%, bringing about a notable increase in ultimate bearing capacity of 33.06% compared with Specimen L2-1, indicating a good reinforcement effect of this method; the SMA wires for Specimen L8-1 had a pre-strain of 3%, and this specimen achieved the highest ultimate bearing capacity among all test specimens (16.7 kN). Although this result is encouraging, the limitations of single-specimen testing and the presence of material defects (e.g., a knot near the crack edge in L8-1) preclude a definitive conclusion regarding the quantitative efficacy of 3% prestressing.

Limitations regarding interpretation of wire count effects: It must be emphasised that, owing to the exploratory nature of this study and the practical difficulties in preparing large-dimension Korean pine beams with controlled crack geometry, only one specimen was tested for each reinforcement configuration. This sample size is insufficient to conduct rigorous statistical analysis, especially given that the bending strength of timber typically exhibits a coefficient of variation of 15% to 25% [31,32]. Furthermore, natural growth defects such as knots were present in several specimens: L4-1 had a knot in the mid-span compression zone; L6-1 had a knot above the crack end; and L8-1 had a knot near the crack edge. These defects, which were not controlled variables in this study, may have individually influenced the ultimate load-bearing capacity and failure behaviour of the beams. Consequently, the observed differences between specimens reinforced with different numbers of SMA wires (L3-1, L4-1 and L5-1) should be interpreted as preliminary trends rather than definitive quantitative conclusions. Future work will involve testing multiple replicates per condition to enable statistical validation and to isolate the effects of material variability from those of the reinforcement scheme.

### 3.2. Load–Deflection Curves

**Figure 30:**
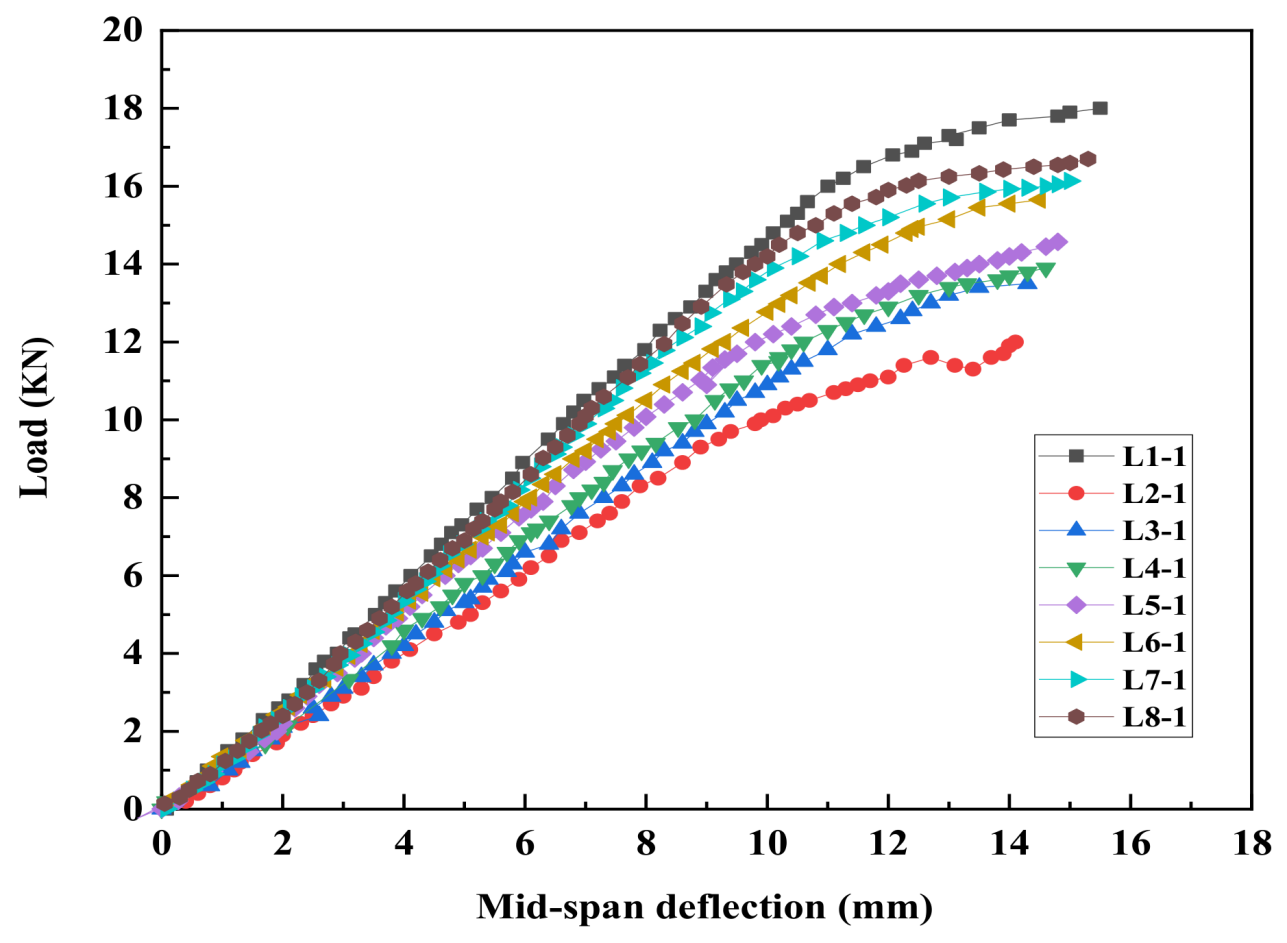
Comparison of load–deflection curves for all test specimens.

**Figure 31:**
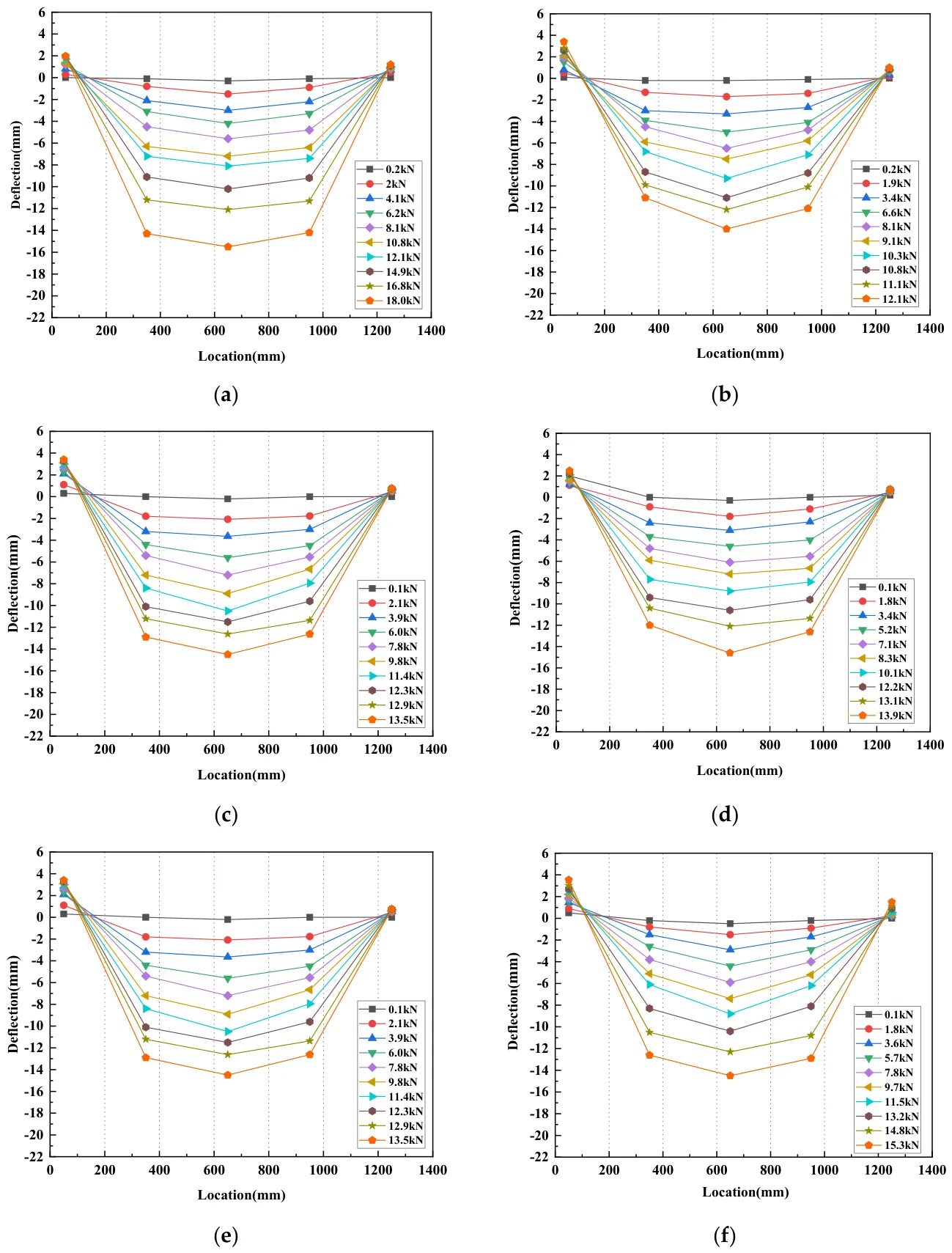

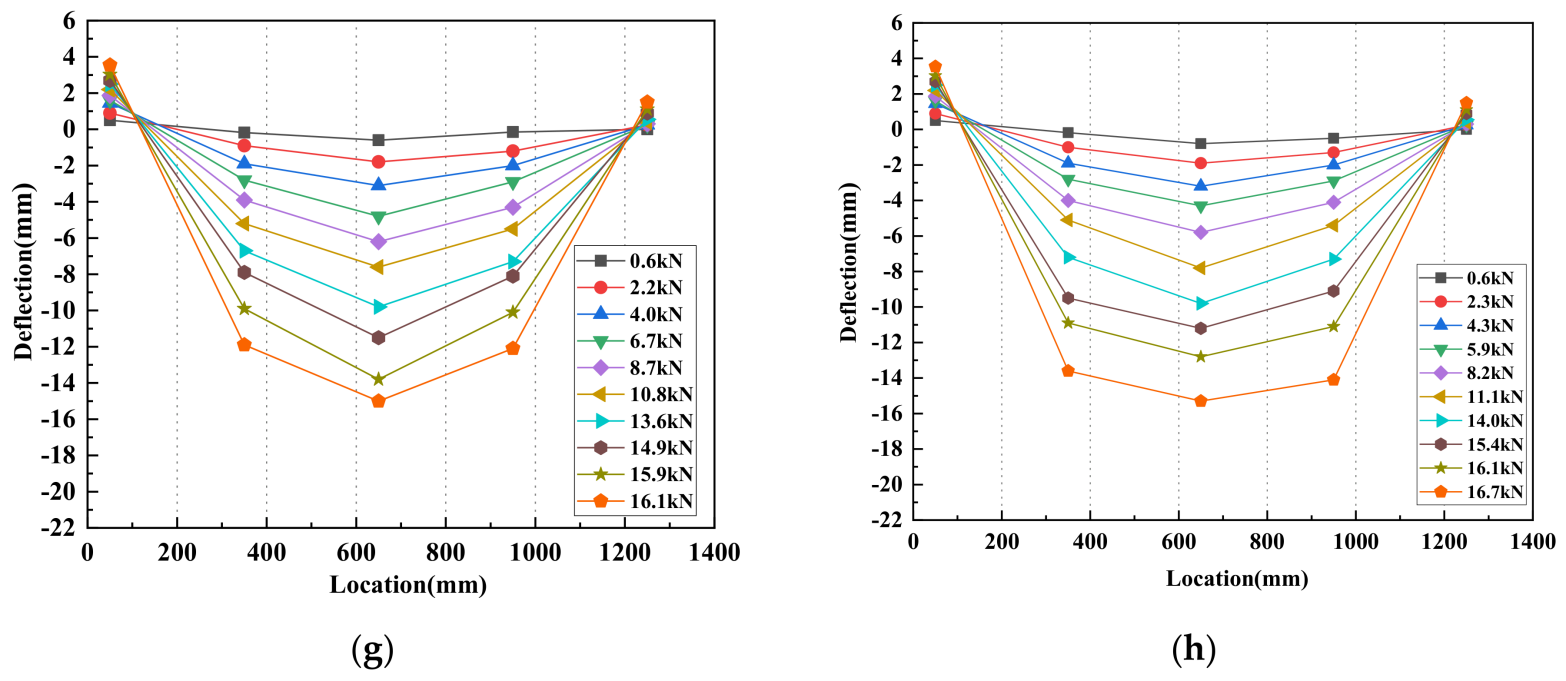
Comparison of load–measurement point–vertical deflection curves for all test specimens. (**a**) L1-1; (**b**) L2-1; (**c**) L3-1; (**d**) L4-1; (**e**) L5-1; (**f**) L6-1; (**g**) L7-1; (**h**) L8-1.

In accordance with the guidance provided in certain references [33,34,35], prior to the test, displacement transducers were installed on the sides of the timber beams in accordance with the test plan, and deflection was recorded using a strain data logger; a pressure transducer was connected to a jack to simultaneously record load data. Based on this, load–deflection at mid-span curves (as shown in Figure 30) and load–measurement point position–vertical deflection curves (as shown in Figure 31) were plotted for each member.

(1) Characteristics of the Load-Fibre-Centre Deflection Curves

The load–deflection curves for the intact timber beam L1-1 and the cracked timber beam L2-1 both exhibit a two-stage characteristic: initially, during the elastic stage, the curve shows linear growth; subsequently, upon entering the elastoplastic stage, the slope of the curve decreases. The ultimate load of L1-1 corresponds to a deflection of 15.5 mm, with the end-of-elastic-stage load at 14.4 kN and a deflection of 11.8 mm. The corresponding values for L2-1 are 14.1 mm and 9.8 kN (deflection 9.9 mm). The slope of the L1-1 curve is significantly steeper than that of L2-1, indicating a marked reduction in the stiffness of the timber beam following cracking.

For the reinforced timber beam L3-1, the elastic stage extended to 11 kN (deflection 11.8 mm), followed by a brief unloading due to the appearance of micro-cracks, after which it entered the elastoplastic stage with a limit deflection of 14.5 mm. For L4-1, the slope decreased after reaching 12 kN in the elastic stage, with a limit deflection of 14.6 mm. For L5-1, the elastic stage extended to 12.2 kN (deflection 10.1 mm), after which the slope decreased slightly. The slopes of the load–deflection curves for all three beams were greater than that of L2-1, indicating that prestressing with superelastic SMA wires effectively increased stiffness, with the most significant improvement observed in L5-1 (3 SMA wires).

L6-1, L7-1 and L8-1 similarly exhibit a two-stage characteristic, with the elastic–plastic transition points occurring at loads of 14 kN, 14.2 kN and 14.8 kN, respectively, and deflections of 11.2 mm, 10.3 mm and 10.5 mm, respectively. The slopes of the L6-1 and L7-1 curves are significantly greater than those of the unreinforced beam, whilst the slope of the elastic segment for L8-1 is close to that of the intact beam. This indicates that adding two SMA wires with 1–3% prestressing to the bottom of the beam can increase the stiffness of the cracked beam; specifically, a 3% prestressing level restores the stiffness to that of the intact beam. In particular, the area under the load–deflection curve prior to reaching the ultimate load represents the beam’s energy absorption capacity. Specimen L8-1 had the largest area, indicating that it possessed the highest energy dissipation capacity of all the specimens. This is consistent with its highest stiffness and ultimate load values.

(2) Load–measurement point position–vertical deflection relationship curve

In accordance with the test protocol, displacement transducers were installed at the mid-span of the timber beam, at the left and right loading points, and at the supports to record the displacement at each location during the loading process. Load–measurement point–vertical deflection curves were plotted, as shown in Figure 31.

The curves show that the deflections of the various members are symmetrically distributed, with the greatest deflection at the mid-span, gradually decreasing towards the supports, indicating that the bending deformation is most pronounced at the mid-span. The deflections at the supports are smaller, and there is even a slight upward camber. In the early stages of loading, the rates of change in deflection for the various members at the same measurement point are similar, indicating the elastic stage; among the different measurement points, the deflection at the mid-span increases the fastest, with the highest rate of change. In the later stages of loading, the timber beams entered the plastic stage, and the rates of deflection change increased at all measurement points; however, the extent of this increase varied between different members: the cracked timber beams exhibited the greatest increase in the rate of change, accompanied by a sudden jump, indicating that the cracks reduced the coordination of deformation; the intact timber beams showed a uniform change, demonstrating good coordination; and the reinforced timber beams exhibited no obvious sudden changes, indicating that prestressing with superelastic SMA wires effectively enhanced the timber beams’ deformation coordination capability.

In order to quantitatively evaluate the stiffness-enhancing effects of different reinforcement schemes, the secant stiffness Ks is defined as the slope of the load–deflection curve between 10% and 40% of the ultimate load; this range represents the linear elastic stage of the timber beam, during which none of the test specimens exhibited any significant cracking or non-linear behaviour. The secant stiffness is calculated using the following Equation (14):

Ks = (P_2_ − P_1_)/(δ_2_ − δ_1_) (14)

where P_1_ and P_2_ are the load values corresponding to 10% and 40% of the ultimate load, respectively, and δ_1_ and δ_2_ are the corresponding deflections at the centre of the span. Table 20 lists the secant stiffness values for all eight test specimens. Among these, L8-1 exhibited the steepest slope (secant stiffness Ks = 1.41 kN/mm) and the largest envelope area (energy absorption ≈ 115.2 kN·mm), demonstrating the most significant increase in stiffness. Specifically, the stiffness of L8-1 was 42.4% higher than that of the cracked beam L2-1 (0.99 kN/mm), and even 15.6% higher than that of the intact beam L1-1 (1.22 kN/mm), indicating that 3% prestressing not only restores but also enhances the original flexural stiffness.

### 3.3. Load–Strain Curve

In accordance with the test protocol and the guidance provided in Reference [36], strain gauges were affixed to the compression zone, the neutral axis and the tension zone at the mid-span of the timber beam. For timber beams L1-1, L2-1, L4-1 and L5-1, measurement point 1 was located in the compression zone, measurement point 2 at the neutral axis, and measurement point 3 in the tension zone; for timber beams L3-1, L6-1, L7-1 and L8-1, measurement points 1 and 2 were located in the compression zone, measurement point 3 was at the neutral axis, and measurement points 4 and 5 were in the tension zone. During the loading process, strain values at each location were recorded by a strain data logger, and load–strain relationship curves were plotted. In the figure, the tension zone is positive and the compression zone is negative.

**Figure 32:**
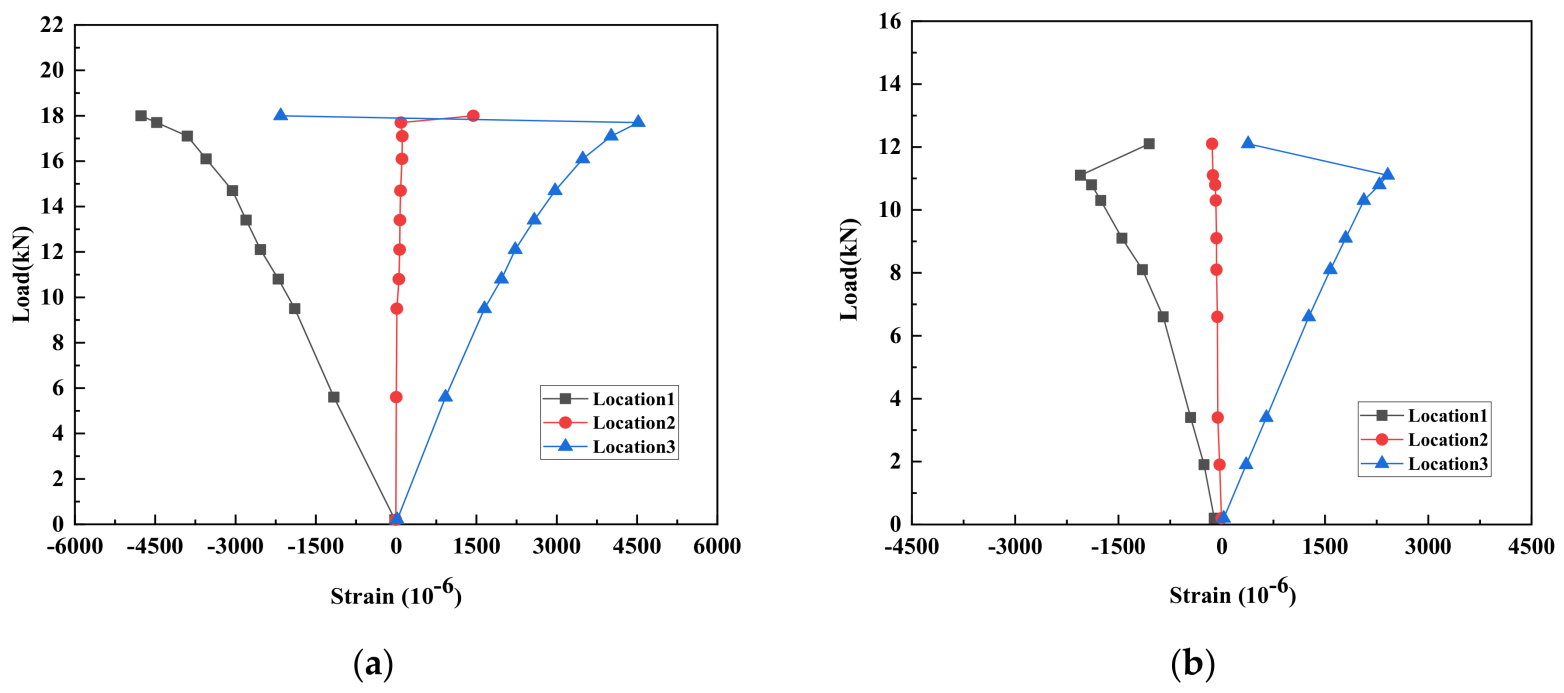
Load–strain curves. (**a**) L1-1; (**b**) L2-1.

As shown in Figure 32, during the loading process, the strain values at the measurement points on the intact timber beam L1-1 and the cracked timber beam L2-1 generally increased linearly with increasing load, and underwent a sudden change upon reaching the ultimate state. At the point of failure, the compressive strains of specimens L1-1 and L2-1 were 4.762 × 10^−3^ and 2.051 × 10^−3^ respectively; the compressive strain of the cracked timber beam was significantly reduced.

**Figure 33:**
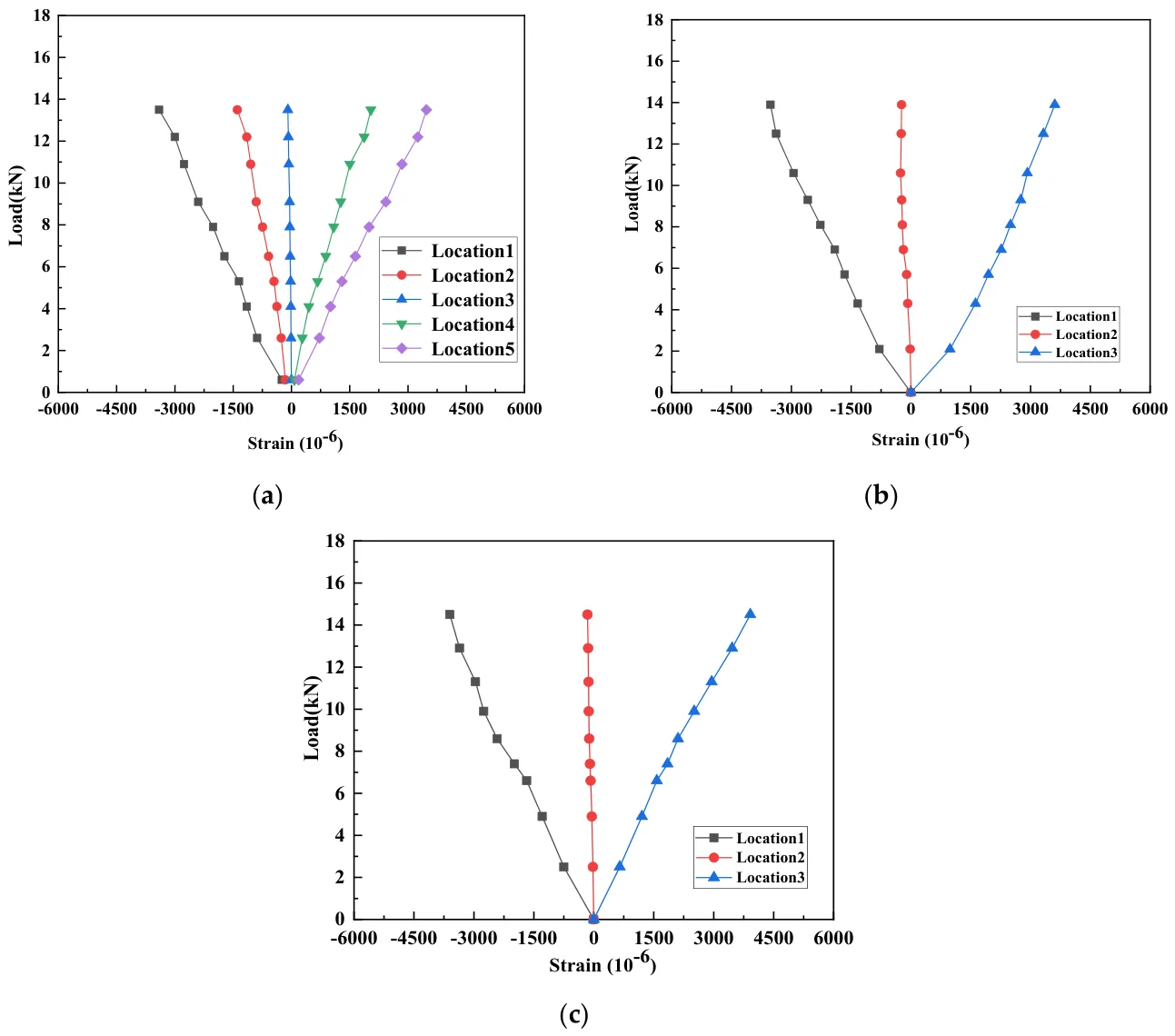
Load–strain curves. (**a**) L3-1; (**b**) L4-1; (**c**) L5-1.

As shown in Figure 33, the strain values of the reinforced timber beams L3-1, L4-1 and L5-1 exhibited a linear variation with increasing load during the initial loading phase. In the later loading phase, distinct non-linear behaviour appeared in both the compression and tension zones; at this point, the timber beams entered the elastic–plastic transition phase, and the rate of change in strain with increasing load slowed down. To evaluate the reinforcement effect on deformation behaviour, the compressive strains of the three beams were compared at a common load level of 10 kN (within the elastic range of all specimens). At this load level, the compressive strains of L3-1, L4-1 and L5-1 were 3.102 × 10^−3^, 2.856 × 10^−3^ and 2.631 × 10^−3^, respectively (values linearly interpolated from the test data). The results show that the compressive strain decreases as the number of SMA wires increases. The fact that the failure compressive strains of these beams (3.406 × 10^−3^, 3.523 × 10^−3^ and 3.602 × 10^−3^, respectively) increase with the number of wires is merely a consequence of the higher ultimate loads achieved by the more heavily reinforced beams, rather than indicating inferior deformation performance. Under identical loading conditions, the beam with more SMA wires consistently exhibited lower compressive strain, demonstrating improved flexural stiffness and enhanced deformation performance.

**Figure 34:**
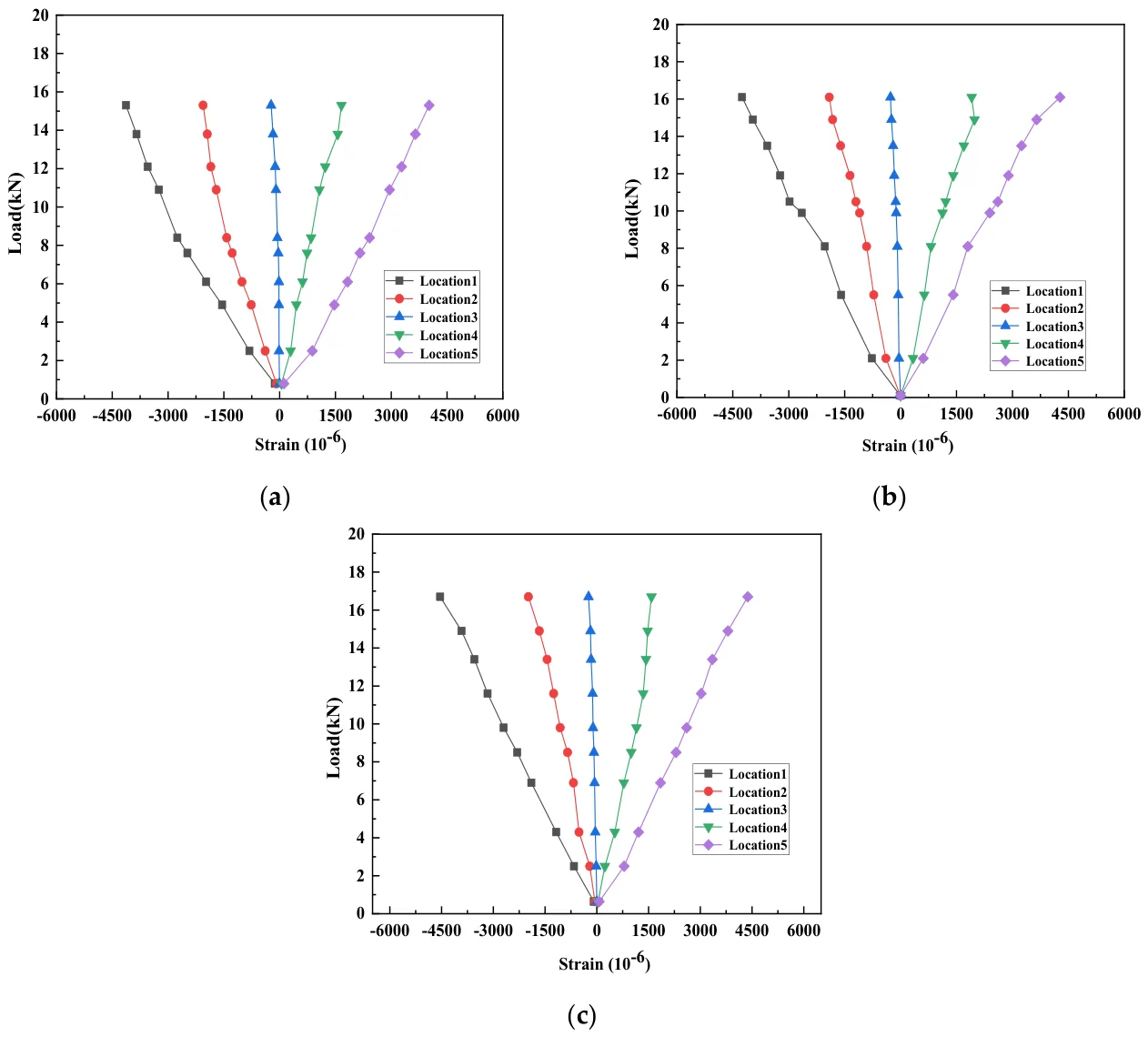
Load–Strain curves. (**a**) L6-1; (**b**) L7-1; (**c**) L8-1.

As can be seen from Figure 34, during the initial loading phase, the strain values at the measurement points of the reinforced timber beams L6-1, L7-1 and L8-1 increased linearly with the load. When the reinforced timber beam L6-1 was subjected to a load of approximately 14 kN, the strain ceased to vary linearly with the load, and the beam entered the elastic–plastic stage. The reinforced timber beams L7-1 and L8-1 both exhibited a tendency to deflect when subjected to a load of approximately 15 kN, and the beams entered the elastic–plastic stage. The maximum compressive strains at failure for the reinforced timber beams L6-1, L7-1 and L8-1 were 4.132 × 10^−3^, 4.251 × 10^−3^ and 4.541 × 10^−3^, respectively, which increased with the degree of prestressing—again reflecting the higher ultimate loads achieved with higher prestressing levels. For a more meaningful comparison, the compressive strains of the three beams were evaluated at a common load level of 12 kN (the highest load level that all three beams sustained in the elastic range). At this load level, the compressive strains of L6-1, L7-1 and L8-1 were 3.512 × 10^−3^, 3.105 × 10^−3^ and 2.824 × 10^−3^, respectively. These results demonstrate that, under identical loading conditions, higher prestressing levels lead to significantly lower compressive strains, indicating improved stiffness and enhanced deformation performance.

### 3.4. Cross-Section–Strain Relationship Curve

**Figure 35:**
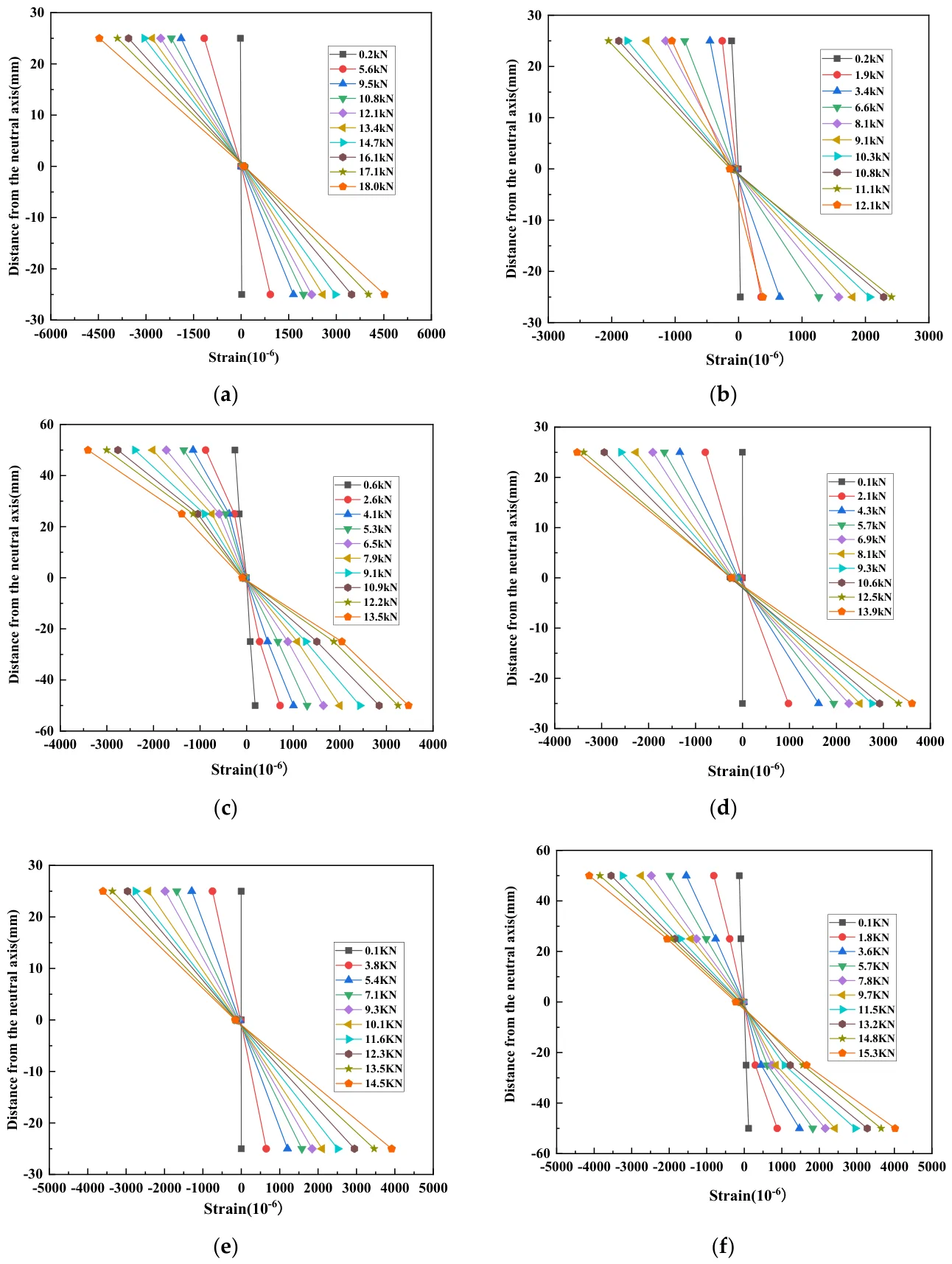

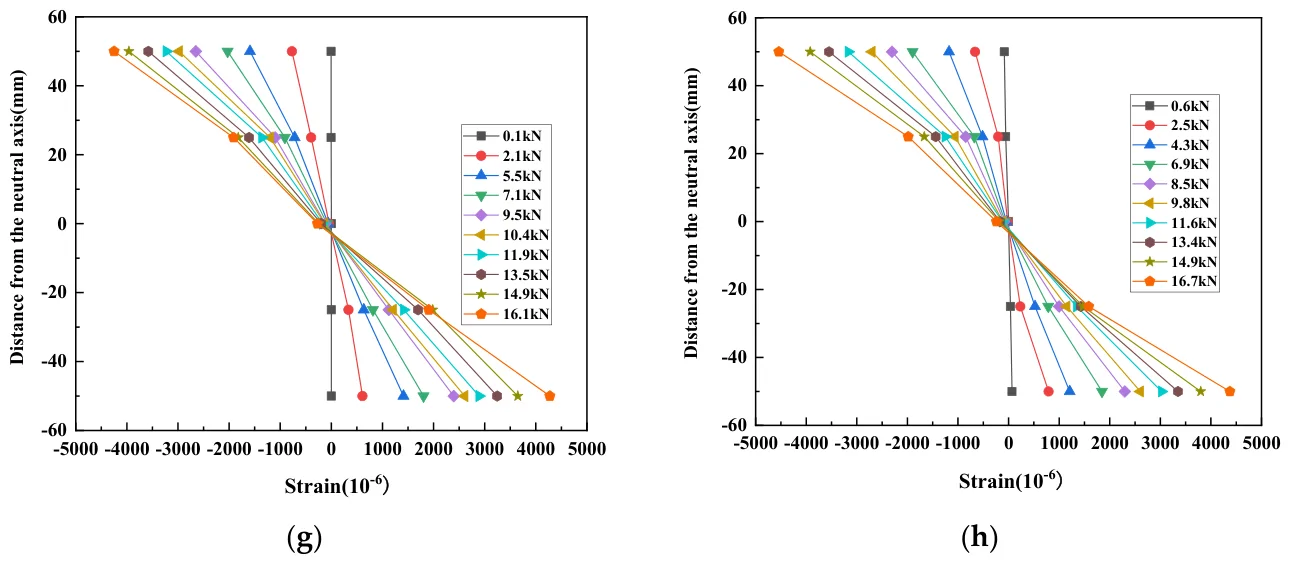
Strain distribution along the cross-section depth at mid-span for all test specimens. (**a**) L1-1; (**b**) L2-1; (**c**) L3-1; (**d**) L4-1; (**e**) L5-1; (**f**) L6-1; (**g**) L7-1; (**h**) L8-1.

In accordance with the test protocol and the guidance provided in References [37,38], strain gauges were affixed at various heights within the mid-span of the timber beam. Strain values were recorded at these different heights under various load levels, and a mid-span cross-section–strain curve was plotted, as shown in Figure 35.

The test results indicate that, under progressively increasing loads, regardless of whether the timber beam is in its initial intact state, a pre-set cracked condition, or a state following SMA prestressing reinforcement, the distribution of mid-span cross-sectional strain along the beam height approximately satisfies the assumption of a flat cross-section, exhibiting good linear and coordinated deformation characteristics. A further comparison between the intact beam L1-1 and the cracked beam L2-1 reveals that the position of the neutral axis in the reinforced beam shifts significantly downwards as the number of SMA strands increases and their prestressing level rises; this change intuitively reflects the effective increase in the height of the compressed zone within the cross-section [39]. From a mechanical perspective, the pre-compressive stress introduced by the SMA prestressing tendons not only partially offsets the unfavourable stresses in the tension zone but also, by adjusting the distribution of internal forces within the cross-section, enhances the contribution of the timber in the compression zone, thereby increasing the cross-section’s equivalent flexural stiffness and ultimate load reserve. The downward shift of the neutral axis implies a more rational redistribution of the areas in the tension and compression zones of the cross-section; the high-strength characteristics of the SMA tendons in the tension zone are fully utilised, whilst the compressive stress level in the timber of the compression zone is maintained within the elastic range, thereby significantly improving the material utilisation and collaborative performance of the cross-section as a whole. In summary, by altering the strain gradient within the cross-section, this reinforcement strategy optimises the load-bearing behaviour of the beam to a certain extent, thereby providing a clear mechanical basis for the design of timber structure reinforcement.

It should be emphasised that, although conventional prestressing tendons (steel) and FRP strips can also improve the ultimate load-bearing capacity of timber beams, SMA wires offer distinct advantages for the retrofitting of existing timber structures—particularly historic buildings. The superelasticity of SMA wires provides a self-centring effect that minimises residual deformation after seismic or overload events, a feature not shared by steel tendons or FRP. Additionally, the excellent corrosion resistance of Ni–Ti alloys ensures long-term durability without the need for protective coatings or regular maintenance, which is highly desirable for heritage structures [40]. The small diameter of SMA wires enables their installation in narrow grooves cut into the beam surface, thereby preserving the original appearance and requiring only minimal intervention—a critical requirement in conservation practice [40]. From a practical standpoint, the prestressing force can be applied using a compact turnbuckle device without heavy hydraulic equipment, making the system suitable for confined or sensitive working environments. The anchorage is achieved via stainless steel plates bolted to the beam ends, which allows for easy inspection and, if necessary, re-tensioning during routine maintenance [41]. This combination of structural efficiency, durability, reversibility, and minimal invasiveness makes SMA wires a particularly attractive option for the reinforcement of diagonally notched timber beams in existing buildings.

It should be noted that the mechanical properties of timber used in this study were all measured from small-sized clear timber specimens in accordance with the Chinese National Standards (GB/T 1927 series [18,19,20,21,22,23,24,25,26]), whilst the test beams were full-scale structural members. The size effect in timber has been well documented in the literature; that is, the strength of structural-scale members is typically lower than that of small-sized clear timber specimens, due to the probabilistic distribution of growth defects, stress concentrations and weak links in the former. However, the primary objective of this study is to evaluate the relative improvement in flexural performance (ultimate load-bearing capacity, stiffness and strain distribution) achieved through prestressing with superelastic SMA wires, rather than to determine the absolute strength of the timber itself. As all reinforced beams were compared with unreinforced cracked beams (L2-1) and intact beams (L1-1) of the same batch and dimensions, the relative values of reinforcement efficiency (e.g., a 38.02% increase in ultimate load-bearing capacity) are valid and unaffected by the size effect. For direct engineering applications, it is recommended to apply appropriate scale factors (e.g., as per ASTM D1990 or EN 384) [42,43] for calibration. Nevertheless, the results of this test have clearly demonstrated the effectiveness of SMA wire reinforcement, and the observed patterns (the effects of wire count and prestressing) are expected to remain qualitatively consistent in larger-scale members. Future research will be extended to full-scale beams of different cross-sectional dimensions in order to quantify the influence of the scale effect on the reinforcement efficiency.

It should also be noted that this study has certain limitations regarding the number of test specimens. Owing to the exploratory nature of this work and the practical constraints involved in machining large-dimension Korean pine beams, only one specimen was tested under each test condition; consequently, it was not possible to conduct a rigorous statistical validation of the results. Nevertheless, the difference in ultimate load-bearing capacity between the reinforced beams and the control beams was significant (for example, L8-1 showed a 38.02% increase compared with L2-1), and the load–deflection and load–strain responses under different reinforcement schemes exhibited clear and consistent patterns, which have clear physical significance, further confirming the potential of SMA for enhancing the performance of timber structures, in line with the conclusions of previous studies [15,16,17]. Furthermore, the mechanical properties of the SMA filaments have been characterised through repeated cyclic loading tests to ensure material stability; the mechanical properties of the timber were measured according to standard methods from multiple small clear-cut specimens (six replicates per test), providing a reliable material basis for comparative analysis. Although the present results are sufficient to demonstrate the feasibility and effectiveness of the proposed reinforcement method, the authors fully recognise that it will be necessary to conduct further tests on additional replicates in the future to clarify the variability and statistical significance of the reinforcement effects.

### 3.5. Prestress Measurement and Loss Evaluation

The initial tensile forces measured after prestressing (prior to the start of the four-point bending test) were as follows: L6-1: 1.83 kN (average per strand). L7-1: 2.41 kN. L8-1: 2.96 kN. The corresponding initial stresses were 808 MPa, 1065 MPa and 1308 MPa, respectively; the 1% and 2% prestressing conditions fell within the expected recovery stress range (500–900 MPa), whilst the 3% condition was slightly above the upper limit. This deviation may be attributed to the combined effects of the actual superelastic behaviour of the SMA wires at the selected prestressing levels, as well as the measurement uncertainty arising from the application of strain gauges to the surface of the 1.2 mm small-diameter wires.

Pre-stress loss was assessed by comparing the initial force measured immediately after tensioning with the force measured before the start of loading (after a 10 min rest period). The measured losses were as follows: L6-1: 4.2%. L7-1: 5.8%. L8-1: 7.3%. These losses were primarily attributed to anchorage slip (at the junction between the stainless steel plate and the fixture) and the elastic shortening of the timber beam following the removal of the tensioning frame. During the subsequent bending loading process, the tensile force in the SMA wire increased monotonically with the external load, indicating that the SMA wire continuously and effectively provided tensile resistance to bridge the crack throughout the test.

It must be acknowledged that the use of strain gauges on 1.2 mm diameter SMA wires has inherent limitations, including possible bending effects and adhesive creep. Future research should consider integrating force transducers into the anchorage system or utilising fibre-optic sensors to achieve more precise force measurements.

The effectiveness of prestressed shape memory alloys (SMAs) in restoring the load-bearing capacity of damaged structures has recently been further confirmed in numerical studies of reinforced concrete (RC) beams. For example, a numerical study on RC beams with voids demonstrated that the application of prestressed iron-based shape memory alloy (Fe-SMA) reinforcement can effectively mitigate the adverse effects caused by voids, restoring up to 95% of the ultimate load-bearing capacity of a solid beam under optimal parameters [44]. Although this study focused on RC beams rather than timber beams, and utilised Fe-SMA rather than the Ni–Ti alloy employed in the present study, its findings are highly consistent with our experimental observations. This consistency across different materials suggests that the reinforcement mechanism of prestressed SMAs—namely, the introduction of compressive stress to counteract tensile stress and control crack propagation—is a universally effective strategy for reinforcing various types of structural members. The positive results from both studies highlight the potential for future research to explore the use of Fe-SMA as a more cost-effective alternative material for reinforcing timber structures.

### 3.6. A Critical Analysis of Experimental Limitations and Uncertainties

As an exploratory study, this research has several inherent limitations regarding the number of test specimens, material control and testing methods, which must be carefully considered. Firstly, due to the difficulty in preparing large-sized Korean pine beams and the requirement for precise control of crack geometry parameters, only a single specimen was tested for each reinforcement scheme; this sample size is insufficient to support rigorous statistical inference. As a natural biomass material, timber exhibits significant inherent variability in its bending strength, ranging from 15% to 25%. Consequently, the differences in ultimate load of 0.4 to 1.0 kN observed between specimens with different numbers of filaments (L3-1, L4-1, L5-1) may be partly attributable to the material’s inherent variability rather than substantive differences in the reinforcement schemes; verification of the relevant trends awaits subsequent repeat experiments. Secondly, natural knots were present in an uncontrollable manner in several test specimens, such as the knot in the mid-span compression zone of L4-1, the knot above the crack tip in L6-1, and the knot at the crack edge in L8-1. Each of these defects may have influenced the ultimate load-bearing capacity, the crack initiation location and the failure mode. In particular, the failure of L8-1 was actually triggered by a knot rather than an artificial notch; its ultimate load of 16.7 kN may represent a conservative lower limit controlled by the defect, failing to fully reflect the true strengthening potential of the 3% prestressing scheme. Furthermore, whilst the use of saw-cut rectangular notches to simulate cracks ensured the repeatability and consistency of the tests, the complete severing of the wood fibres—and the elimination of fibre bridging and interlocking effects—represents a fundamental difference from the naturally aged oblique cracks found in actual historic timber beams, which feature irregular surfaces and overlapping fibres. This may result in a conservative assessment of the crack bridging efficiency of SMA wires; therefore, caution must be exercised when extrapolating quantitative values to actual aged structures.

With regard to the generalisability of the measurement methods and results, the tensile force of the SMA wire is calculated using strain gauges affixed to the surface of the 1.2 mm diameter wire; this method has inherent limitations, such as the bending effect of the fine wire, creep of the adhesive layer and local strain disturbances. The measured initial stress value (1308 MPa) under 3% prestressing conditions exceeded the supplier’s specified recovery stress range (500–900 MPa); this deviation stems, at least in part, from measurement uncertainty. In future, micro-load cells or fibre-optic gratings should be integrated to obtain more reliable tensile force data. Furthermore, all tests were conducted exclusively on Korean pine (*Pinus koraiensis*) and cross-sections measuring 80 mm × 100 mm. As a typical softwood, Korean pine has a ratio of longitudinal to transverse strength of approximately 9:1; the effectiveness of this reinforcement method requires further validation when applied to hardwoods or significantly different cross-sectional dimensions. Potential sources of uncertainty also include the accuracy of manual loading control, minute variations in moisture content between specimens (although these were maintained within the standard range of 9–15%), and approximation errors introduced by linear interpolation of compressive strain during the elastic stage. In summary, this exploratory study provides sufficient evidence to demonstrate the feasibility and effectiveness of reinforcing diagonally cracked timber beams with prestressed SMA wires; however, it is not yet possible to quantify statistical significance. There is an urgent need for follow-up studies involving multiple replicate specimens, utilising high-precision force measurement techniques and covering a wider range of material types to validate and extend the conclusions presented in this paper.

## 4. Conclusions

To investigate the effectiveness of different reinforcement methods, four-point bending tests were conducted on eight timber beams (including intact beams, cracked beams and six reinforced beams). The failure behaviour, ultimate load-carrying capacity, load–deflection curves and load–strain curves were analysed. Based on an examination of limit states, stiffness and strain, the following conclusions were drawn:

(1) The installation of SMA wires on the bottom of the beam effectively increases the ultimate load-bearing capacity of the timber beam. The reinforced beams with three SMA wires showed the most significant improvement, with a 19.83% increase in load-bearing capacity compared to the cracked beams; those with one wire showed an 11.57% increase. The load-bearing capacities of the reinforced beams subjected to prestressing (L6-1, L7-1, L8-1) increased by 26.45%, 33.06% and 38.02% respectively, which were higher than those of the non-prestressed reinforced beams. Furthermore, the extent of improvement increased with the magnitude of prestressing, with L8-1 (3% prestressing) showing the greatest improvement.
(2) The load–deflection curves of the reinforced timber beams were all divided into elastic and elastoplastic stages. Although the slopes of the curves differed under different reinforcement methods, they were all greater than those of the cracked beams, indicating an improvement in deformation performance. Among these, L8-1 exhibited the steepest slope and the largest envelope area, demonstrating the most significant increase in stiffness.
(3) SMA wire reinforcement causes the neutral axis of the timber beam to shift downwards, resulting in an expansion of the compression zone and a reduction in the tension zone, thereby enhancing deformation capacity. Under identical loading conditions, the reinforced timber beam with two SMA wires pre-stressed to 3% (L8-1) exhibits the lowest compressive strain and the most significant improvement in flexural stiffness among all reinforced specimens. This confirms that higher prestressing levels are more effective in improving the deformation performance of diagonally cracked timber beams.

In addition to the above experimental findings, it is worth highlighting that SMA wires offer significant practical advantages over conventional steel tendons or FRP strips for the reinforcement of existing timber buildings. Their superelasticity provides an intrinsic self-centring capability that reduces residual deformations after extreme loading events. Their excellent corrosion resistance eliminates the need for regular maintenance, making them particularly suitable for heritage structures where long-term durability is essential. The small wire diameter allows for minimally invasive installation, and the anchorage system can be designed to be reversible, which is often a mandatory requirement in conservation projects. These features, combined with the structural improvements demonstrated in this study, support the use of SMA wires as a viable and advantageous reinforcement solution for diagonally cracked timber beams in existing structures. Future research should investigate the long-term performance of SMA-reinforced timber beams under environmental loading, as well as the optimisation of anchorage details for different beam geometries and crack patterns. Furthermore, given the single-specimen design of this exploratory study, future work should include multiple replicate specimens per test condition to enable statistical validation of the reinforcement efficiency and to isolate the effects of inherent material variability.

## Figures and Tables

**Table 1:** Key properties of the Ni–Ti SMA wires.

Parameter	Value
Supplier	Dongguan Wuhang Metal Materials Co., Ltd. (Dongguan, China)
Material type	Ni–Ti shape memory alloy (superelastic)
Wire diameter	1.2 mm
Cross-sectional area	1.131 mm^2^
Chemical composition (wt%)	Ni 56.2%, Ti 43.76% (balance), O 0.026%, H 0.001%, C 0.0034%
Austenite finish temperature	<35 °C (confirmed by the supplier; ensuring superelasticity at room temperature)
Martensite finish temperature	Greater than or equal to −45 °C
Superelastic recovery strain	8%
Recovery stress	500–900 MPa
Elastic modulus (austenite)	Approximately 83 GPa
Elastic modulus (martensite)	28–41 GPa
Ultimate tensile strength	1000–1300 MPa
Density	6.45 g/cm^3^

**Table 2:** Summary of moisture content measurements for all mechanical property tests (%).

Test Type	Specimen Count	MC Range	Average MC
Compressive strength (parallel)	6	10.8–12.1	11.5
Compressive strength (radial)	6	10.5–12.2	11.4
Compressive strength (tangential)	6	10.6–12.0	11.3
Tensile strength (parallel)	6	10.9–12.5	11.7
Tensile strength (radial)	6	10.5–12.1	11.3

**Table 3:** Compressive strength of Korean pine (*Pinus koraiensis*) along the grain (MPa).

Specimen Number	Test Strength	Corrected Strength	Average Corrected Strength
1	39.3	31.3	31.3
2	38.5	30.6	
3	37.7	30.0	
4	41.4	32.9	
5	40.5	32.4	
6	38.5	30.6	

**Table 4:** Radial compressive strength of Korean pine (MPa).

Specimen Number	Test Strength	Corrected Strength	Average Corrected Strength
1	4.3	3.5	3.6
2	4.6	3.8	
3	4.5	3.6	
4	4.2	3.5	
5	4.4	3.6	
6	4.7	3.8	

**Table 5:** Tangential compressive strength of Korean pine (MPa).

Specimen Number	Test Strength	Corrected Strength	Average Corrected Strength
1	4.8	3.9	3.9
2	4.6	3.8	
3	4.1	4.1	
4	4.9	4.0	
5	4.4	3.6	
6	4.8	3.9	

**Table 6:** Compressive modulus of elasticity of Korean pine (MPa).

Specimen Number	Compressive Modulus of Elasticity	Corrected Compressive Modulus of Elasticity	Average Corrected Compressive Modulus of Elasticity
1	9691.5	9214.7	9898.3
2	9435.4	8971.1	
3	11,035.2	10,492.3	
4	11,778.9	11,199.4	
5	9581.5	9110.1	
6	10,940.8	10,402.5	

**Table 7:** Modulus of elasticity for radial compression of Korean pine (MPa).

Specimen Number	Compressive Modulus of Elasticity	Corrected Compressive Modulus of Elasticity	Average Corrected Compressive Modulus of Elasticity
1	2139.7	1657.2	1521.5
2	1917.7	1485.3	
3	1770.4	1371.1	
4	2287.6	1771.7	
5	1945.2	1506.5	
6	1726.8	1337.4	

**Table 8:** Modulus of elasticity for tangential compression of Korean pine (MPa).

Specimen Number	Compressive Modulus of Elasticity	Corrected Compressive Modulus of Elasticity	Average Corrected Compressive Modulus of Elasticity
1	986.0	763.6	762.6
2	984.6	762.6	
3	976.7	756.5	
4	953.6	738.6	
5	1018.0	788.4	
6	988.7	765.8	

**Table 9:** Tensile strength of Korean pine along the grain (MPa).

Specimen Number	Test Strength	Corrected Strength	Average Corrected Strength
1	58.9	55.3	53.1
2	55.9	52.5	
3	57.3	53.8	
4	57.1	53.6	
5	54.0	50.6	
6	56.1	52.6	

**Table 10:** Tensile strength in the radial direction of Korean pine (MPa).

Specimen Number	Test Strength	Corrected Strength	Average Corrected Strength
1	4.2	3.8	3.8
2	4.0	3.6	
3	4.5	4.0	
4	4.3	3.8	
5	4.2	3.8	
6	4.0	3.6	

**Table 11:** Tensile strength in the tangential direction of Korean pine (MPa).

Specimen Number	Test Strength	Corrected Strength	Average Corrected Strength
1	3.5	3.1	3.2
2	3.4	3.0	
3	3.2	2.9	
4	3.9	3.5	
5	3.6	3.2	
6	3.7	3.4	

**Table 12:** Modulus of elasticity in bending for Korean pine (MPa).

Specimen Number	Test Flexural Modulus of Elasticity	Corrected Flexural Modulus of Elasticity	Average Corrected Flexural Modulus of Elasticity
1	11,588.1	10,875.4	9690.8
2	9575.6	8986.7	
3	10,382.3	9743.8	
4	10,114.0	9492.0	
5	9804.0	9201.0	
6	10,490.3	9845.8	

**Table 13:** Flexural strength of Korean pine (MPa).

Specimen Number	Test Strength	Corrected Strength	Average Corrected Strength
1	112.2	93.8	91.6
2	97.6	81.6	
3	108.2	90.5	
4	120.9	101.1	
5	110.5	92.4	
6	108.3	90.5	

**Table 14:** Shear strength of Korean pine in the tangential plane (MPa).

Specimen Number	Test Strength	Corrected Strength	Average Corrected Strength
1	4.6	4.0	4.2
2	4.8	4.2	
3	4.9	4.3	
4	5.1	4.4	
5	4.9	4.3	
6	4.7	4.1	

**Table 15:** Shear strength of Korean pine in the radial plane.

Specimen Number	Test Strength	Corrected Strength	Average Corrected Strength
1	20.9	18.3	16.8
2	18.2	16.0	
3	18.5	16.2	
4	19.6	17.2	
5	20.0	17.6	
6	17.8	15.6	

**Table 16:** Estimated shear modulus of Korean pine (MPa).

Shear Modulus	Value
G_rl_ (radial–longitudinal)	618.6
G_tl_ (tangential–longitudinal)	549.9
G_rt_ (radial–tangential)	190.2

**Table 17:** Parameter settings for test timber beams.

Specimen Number	Member Dimensions (mm)	Crack Dimensions (mm)	Number of SMA Strands	SMA Strand Prestress
L1-1	1300 × 80 × 100	-	-	-
L2-1	1300 × 80 × 100	190 × 10 × 20	-	-
L3-1	1300 × 80 × 100	190 × 10 × 20	1	-
L4-1	1300 × 80 × 100	190 × 10 × 20	2	-
L5-1	1300 × 80 × 100	190 × 10 × 20	3	-
L6-1	1300 × 80 × 100	190 × 10 × 20	2	1%
L7-1	1300 × 80 × 100	190 × 10 × 20	2	2%
L8-1	1300 × 80 × 100	190 × 10 × 20	2	3%

**Table 18:** Summary of failure modes and key characteristics for all test specimens.

Specimen	Reinforcement Method	Failure Mode Description	Key Characteristics/Observations	Figure Reference
L1-1	Intact beam (no crack, no SMA)	Brittle tension failure in pure bending zone.	Initiated at the left loading point; single main crack propagated at a 40° angle from the bottom towards mid-span; sudden loud bang with wood shavings ejected; no warning signs before failure.	Figure 15
L2-1	Cracked beam (artificial notch, no SMA)	Brittle failure triggered by the artificial notch.	Crack initiated at the notch tip in the right shear-bending zone; propagated rapidly along the 6° inclined notch direction; sudden fracture with a loud bang; complete loss of load capacity at failure.	Figure 17
L3-1	1 SMA wire (0% prestress)	Crack propagation failure from the notch, partially bridged by SMA.	Single SMA wire provided limited crack-bridging resistance; cracking sounds heard from the notch tip at 6.8 kN; final fracture along the notch direction at 13.5 kN; SMA wire remained intact but was not fully embedded into the timber.	Figure 19
L4-1	2 SMA wires (0% prestress)	Controlled crack propagation with effective crack bridging.	Two SMA wires bridged the crack, preventing sudden beam snap; failure accompanied by a loud bang at 13.9 kN; SMA wires remained in place and continued to function after timber fracture, showing a more ductile failure process compared to L3-1.	Figure 21
L5-1	3 SMA wires (0% prestress)	Compression-dominated failure with multiple crack bridging.	Three SMA wires provided the best crack control among non-prestressed groups; crack propagation was gradual; final failure at 14.5 kN showed wood fibre crushing in the compression zone; SMA wires remained taut throughout the test.	Figure 23
L6-1	2 SMA wires (1% prestress)	Delayed crack initiation with effective prestress effect.	Prestress delayed crack opening; first cracking sound at 7.1 kN; failure at 15.3 kN with fibres torn at the crack base; SMA wires remained highly stressed; failure mode showed a transition towards compression zone crushing.	Figure 25
L7-1	2 SMA wires (2% prestress)	Compression-zone crushing failure; tension-zone effectively strengthened.	Prestress significantly improved crack control; no visible surface cracks until 10.4 kN; failure at 16.1 kN characterised by wood crushing in the top compression zone; SMA wires fully mobilised; bending deformation clearly visible before failure.	Figure 27
L8-1	2 SMA wires (3% prestress)	Defect-triggered compression failure near a natural knot.	Highest prestress level; failure initiated at a natural knot near the crack edge rather than at the notch; cracking sound at the knot at 11.1 kN; final failure at 16.7 kN with crack extension from the knot; demonstrated that tension zone reinforcement was so effective that failure was governed by other material defects.	Figure 28

**Table 19:** Bearing capacity data of timber beams.

Specimen Number	Reinforcement Method	Ultimate Bearing Capacity/kN	Increase Rate vs. Damaged Timber Beam
L1-1	None	18	-
L2-1	None	12.1	-
L3-1	1 SMA wire with 0% pre-strain	13.5	11.57%
L4-1	2 SMA wires with 0% pre-strain	13.9	14.88%
L5-1	3 SMA wires with 0% pre-strain	14.5	19.83%
L6-1	2 SMA wires with 1% pre-strain	15.3	26.45%
L7-1	2 SMA wires with 2% pre-strain	16.1	33.06%
L8-1	2 SMA wires with 3% pre-strain	16.7	38.02%

**Table 20:** Secant stiffness of all test specimens.

Specimen	Pult (kN)	P_1_ (10%) (kN)	P_2_ (40%) (kN)	δ_1_ (mm)	δ_2_ (mm)	Secant Stiffness Ks (kN/mm)	Relative to L1-1
L1-1	18.0	1.80	7.20	1.48	5.90	1.22	1.00
L2-1	12.1	1.21	4.84	1.22	4.89	0.99	0.81
L3-1	13.5	1.35	5.40	1.45	5.81	0.93	0.76
L4-1	13.9	1.39	5.56	1.32	5.30	1.05	0.86
L5-1	14.5	1.45	5.80	1.20	4.79	1.21	0.99
L6-1	15.3	1.53	6.12	1.22	4.90	1.25	1.02
L7-1	16.1	1.61	6.44	1.17	4.67	1.38	1.13
L8-1	16.7	1.67	6.68	1.18	4.74	1.41	1.16

## Data Availability

The original contributions presented in this study are included in the article. Further inquiries can be directed to the corresponding authors.
